# Multiple Sclerosis: Lipids, Lymphocytes, and Vitamin D

**DOI:** 10.20900/immunometab20200019

**Published:** 2020-05-07

**Authors:** Colleen E. Hayes, James M. Ntambi

**Affiliations:** 1 Department of Biochemistry, College of Agricultural and Life Sciences, University of Wisconsin-Madison, 433 Babcock Drive, Madison, WI 53706, USA; 2 Department of Nutritional Sciences, University of Wisconsin-Madison, 1415 Linden Drive, Madison, WI 53706, USA

**Keywords:** multiple sclerosis, vitamin D, obesity, oligodendrocytes, myelin, nervonic acid, T lymphocytes, methionine cycle, epigenetic regulation

## Abstract

Multiple sclerosis (MS) is an inflammatory demyelinating disease of the central nervous system. We review the two core MS features, myelin instability, fragmentation, and remyelination failure, and dominance of pathogenic CD4^+^ Th17 cells over protective CD4^+^ Treg cells. To better understand myelin pathology, we describe myelin biosynthesis, structure, and function, then highlight stearoyl-CoA desaturase (SCD) in nervonic acid biosynthesis and nervonic acid’s contribution to myelin stability. Noting that vitamin D deficiency decreases SCD in the periphery, we propose it also decreases SCD in oligodendrocytes, disrupting the nervonic acid supply and causing myelin instability and fragmentation. To better understand the distorted Th17/Treg cell balance, we summarize Th17 cell contributions to MS pathogenesis, then highlight how 1,25-dihydroxyvitamin D_3_ signaling from microglia to CD4^+^ T cells restores Treg cell dominance. This signaling rapidly increases flux through the methionine cycle, removing homocysteine, replenishing S-adenosyl-methionine, and improving epigenetic marking. Noting that DNA hypomethylation and inappropriate DRB1*1501 expression were observed in MS patient CD4^+^ T cells, we propose that vitamin D deficiency thwarts epigenetic downregulation of DRB1*1501 and Th17 cell signature genes, and upregulation of Treg cell signature genes, causing dysregulation within the CD4^+^ T cell compartment. We explain how obesity reduces vitamin D status, and how estrogen and vitamin D collaborate to promote Treg cell dominance in females. Finally, we discuss the implications of this new knowledge concerning myelin and the Th17/Treg cell balance, and advocate for efforts to address the global epidemics of obesity and vitamin D deficiency in the expectation of reducing the impact of MS.

## INTRODUCTION

Elucidating the molecular etiology of neurodegenerative disease remains a major challenge in modern neuroscience [1]. Disturbances in vitamin D metabolism and in brain lipid content have been observed in multiple sclerosis (MS), Alzheimer’s disease, and Parkinson’s disease. Focusing on MS, we provide a perspective on the biochemical, biological, and immunological issues related to vitamin D metabolism, lipid metabolism, and the possible relationships between them as regards the pathogenesis of neurodegenerative disease. MS is an autoimmune demyelinating disease of the central nervous system (CNS) for which there is detailed scientific knowledge about myelin, the target of the autoimmune response. There is also a deep understanding of MS susceptibility and risk genes, predisposing and protective environment exposures, gender and hormonal influences, and the interplay between these factors that ultimately determines MS risk. Increasing knowledge of the molecular etiology of MS will inform interventional strategies aimed at reducing its impact and perhaps the impact of other complex neurodegenerative diseases.

## AN AUTOIMMUNE-MEDIATED DEMYELINATING DISEASE

The precise etiology of MS is unknown, but interactions between heritable MS susceptibility, exposure to environmental risk factors, and sex hormones are believed to contribute to disease development [2,3]. These interactions are believed to initiate pathobiological processes that damage the CNS many years or possibly decades before MS is clinically evident [4–6]. The possibility of reducing MS incidence has motivated a close examination of when and how modifiable environmental risk factors influence the pathobiological processes that damage the myelin sheath. Here, we focus on two well-established non-genetic factors that influence MS pathogenesis in childhood and adolescence, vitamin D insufficiency, and obesity and lipid metabolism.

Neurological dysfunction in MS patients is attributed to inflammatory demyelinating lesions that cause axonal transmission failure, axonal degeneration, and finally, neuronal cell death. In relapsing-remitting MS (RRMS) disease, these lesions partially resolve and some neurological function is recovered, but reactivation of previous lesions, new lesion formation and relapses of neurological dysfunction occur. In contrast, uninterrupted lesion evolution and unrelenting accumulation of neurological impairment characterize progressive MS.

At its core MS is an inflammatory demyelinating disease. The myelin sheath is a lipid-rich structure that wraps axons and facilitates fast neurotransmission within the brain and between the brain and the body. Close examination of MS demyelinating lesions has revealed an inflammatory process that causes CNS tissue injury at all stages of MS disease [7,8]. Early acute lesions show activated microglia, activated T lymphocytes, oligodendrocyte loss, demyelination, myelin-laden macrophages, and reactive astrocytes in focal regions surrounding the perivasculature. Chronic lesions show activated microglia, oligodendrocyte loss, demyelination, axonal damage and loss, and only a few T lymphocytes. These observations clearly suggest immune-mediated damage, but the origins of the inflammatory process are debated.

Immune responses to infectious pathogens depend on CD4^+^ T helper lymphocytes to direct protective effector mechanisms. Pathogen epitope diversity is orders of magnitude larger than T cell receptor (TCR) diversity and TCR molecules exhibit some flexibility, so some cross-reactivity of pathogen-specific CD4^+^ T cells with host tissue epitopes is inevitable [9]. Extensive autoimmune-mediated damage to host tissues can occur if these auto-reactive CD4^+^ T cells persist and become activated.

Myelin-reactive, IL-17A-producing, CD4^+^CCR6^+^ Th17 cells are widely believed to have a fundamental role in MS demyelinating lesion development [10]. These cells have been identified in the peripheral blood and cerebrospinal fluid (CSF) of MS patients [11]. They exhibit a gene expression pattern that conforms to the encephalitogenic signature of CD4^+^ Th17 cells derived from experimental autoimmune encephalomyelitis (EAE) lesions in rodents [12]. Notably, the CD4^+^ Th17 cells express the CCR6 and CXCR3 chemokine receptors and do not produce significant amounts of IL-10.

The CD4^+^ Th17 cells develop in peripheral immune organs in response to stimulation by pathogen epitopes presented by antigen presenting cells (APC) in the context of pro-inflammatory cytokines (IL-6, IL-21, IL-23, and TGF-β) [13,14]. The cytokines induce retinoid receptor-related orphan nuclear receptor gamma-t (RORγt) as the lineage-determining transcription factor for CD4^+^ Th17 cells. The activated CD4^+^ Th17 cells then leave the peripheral immune organs and migrate to sites of infection guided by CCR6 and CXCR3 chemokine receptor ligation. Some myelin-reactive CD4^+^ Th17 cells activated in the periphery gain access to the CNS where they initiate demyelinating lesion formation [9].

Immunological mechanisms have evolved to terminate effector CD4^+^ T cell responses upon pathogen removal, thereby preventing autoimmune-mediated damage to host tissues. Foundational to these mechanisms are the IL-10-producing CD4^+^ T regulatory cells (Treg) [15]. These cells use the *Ikzf2* gene encoding Helios and the *Foxp3* gene encoding FoxP3 as lineage-specifying transcriptional factor genes [16,17]. They also express the high affinity IL-2-receptor (CD25]. The IL-2 provides support for cell survival, proliferation, and suppressive function.

Several mechanisms allow CD4^+^FoxP3^+^ Treg cells to terminate effector CD4^+^ T cell responses upon pathogen removal. They consume the IL-2 that is produced by the effector Th17 cells so growth factor deprivation slows Th17 cell expansion and decreases cell survival. They produce IL-10, IL-35, and TGF-β to inhibit Th17 cell cytokine synthesis [18]. Finally, they express CTLA-4 which strips the CD28-costimulatory ligands CD80 and CD86 from neighboring APC [19]. Depriving the APC of CD80 and CD86 costimulatory molecules suppresses the ability of APC to initiate new effector CD4^+^ T cell activation. Collectively, these and other actions terminate CD4^+^ Th17 cell responses before immune-mediated pathology occurs. In MS and EAE, distortion of the Th17/Treg cell balance in favor of pro-inflammatory CD4^+^ Th17 cells has been demonstrated [12,20]. This distortion is believed to have a causal role in myelin-reactive CD4^+^ Th17 cell-mediated lesion development.

Understanding the genesis of the distorted Th17/Treg cell balance is foundational to our efforts to prevent and treat MS. Importantly, the mature pro-inflammatory CD4^+^ Th17 cells show some instability and functional adaptability [13,14]. Cell fate mapping experiments in the EAE model demonstrated that myelin-specific CD4^+^ Th17 cells permanently marked for their *Il17* gene expression underwent global genetic reprogramming during EAE resolution; they stopped producing IL-17A and started producing IL-10 [21]. This discovery challenges researchers to define the triggers that promote CD4^+^ Th17 cell genetic reprogramming to a CD4^+^ Treg cell phenotype to prevent or limit autoimmune-mediated damage to host tissues. Three T cell intrinsic factors influence the Th17/Treg cell balance, cholesterol biosynthetic intermediate signaling to RORγt in Th17 cells [22], sphingomyelin breakdown and ceramide signaling [23], and paracrine 1,25-(OH)_2_D_3_ signaling to the vitamin D receptor (VDR) in Th17 cells and Treg cells [24]. We focus on 1,25-(OH)_2_D_3_-VDR signaling in this review.

### Epigenetics and Heritability of MS Risk

**Figure 1.**
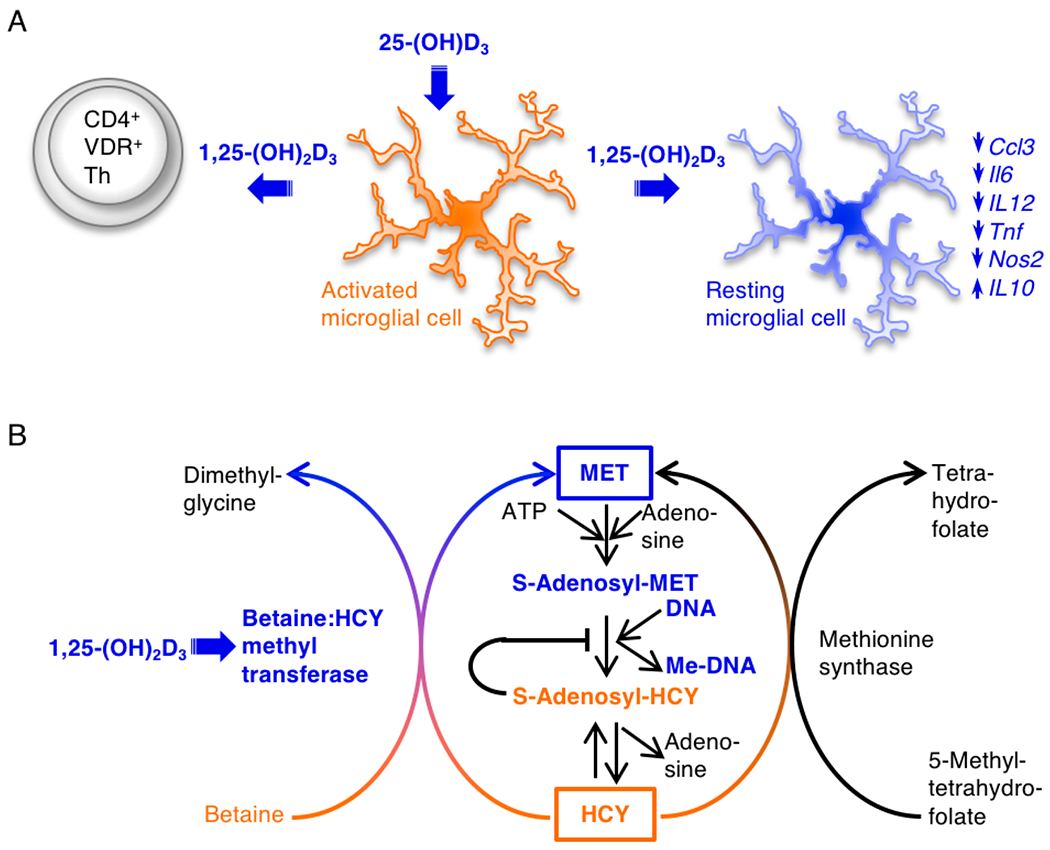

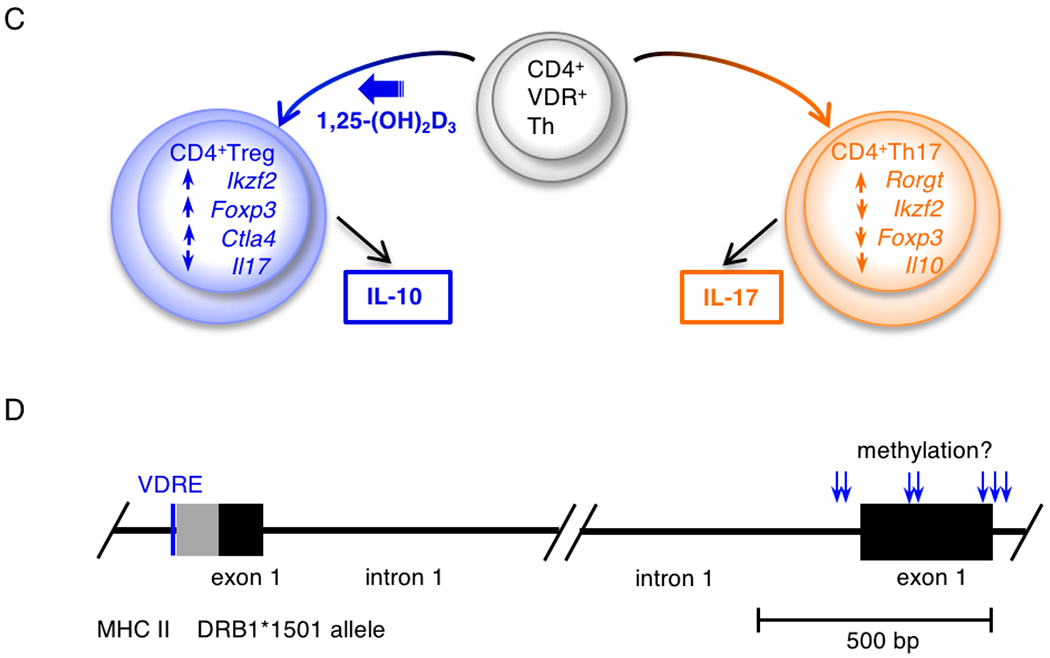
The vitamin D-epigenetic hypothesis of MS risk. (**A**) Myeloid lineage cells produce 1,25-(OH)_2_D_3_ for paracrine VDR signaling to CD4^+^ T cells in the inflamed CNS; in vitamin D deficiency this signal is absent. (**B**) The 1,25-(OH)_2_D_3_-VDR signaling increases metabolite flux through the MET cycle; in vitamin D deficiency insufficient MET cycle activity causes toxic HCY and *S*-adenosyl-HCY to accumulate, blocking DNA methylation. (**C**) The 1,25-(OH)_2_D_3_-VDR signaling promotes *Ikzf2, Foxp3, Ctla4* gene transcription and CD4^+^ Treg cell development while inhibiting *Il17* gene transcription; in vitamin D deficiency *Il17* gene transcription is not repressed, the *Ikzf2, Foxp3*, and *Ctla4* genes are not induced, and the Treg/Th17 balance is distorted in favor of Th17 cells. (**D**) the 1,25-(OH)_2_D_3_-VDR signaling might promote epigenetic downregulation of DRB1*1501 gene transcription; in vitamin D deficiency DRB1*1501 gene transcription might be elevated, favoring Th17 cell development. Adequate vitamin D status might prevent dysregulation within the CD4^+^ T cell compartment. Orange objects and text represent pro-inflammatory processes. Blue objects and text represent proposed protective actions of the vitamin D system.

The major histocompatibility complex (MHC) class II region largely determines the heritable component in MS genetic susceptibility [25,26]. The potential role of epigenetic modifications in MS heritability has been reviewed (see Figure 1 in [27]). We consider DNA methylation in detail, because this epigenetic mechanism confers heritable changes in MHC class II gene expression without altering the underlying DNA sequence [28]. New research has linked DNA hypomethylation within exon 2 of the MHC class II DRB1*1501 MS risk allele, abundant transcripts of this allele in monocytes, B cells, CD4^+^ T cells, and CD8^+^ T cells, and MS disease status [29,30]. It is noteworthy that this allele has a candidate VDRE near the transcription start site. Moreover, animal modeling research has demonstrated paracrine 1,25-dihydroxyvitamin D_3_ (1,25-(OH)_2_D_3_)-vitamin D receptor (VDR) signaling from myeloid lineage cells to CD4^+^ T cells in the CNS [31], 1,25-(OH)_2_D_3_-mediated enhancement of betaine:homocysteine methyltransferase (BHMT1) and metabolite flux through the methionine (MET) cycle, DNA methylation, and CD4^+^Helios^+^FoxP3^+^ Treg cell dominance in EAE [32]. These advances suggest the vitamin D-epigenetic hypothesis of MS risk (Figure 1). High vitamin D status (i) increases paracrine 1,25-(OH)_2_D_3_-VDR signaling from microglia to CD4^+^ T cells; (ii) improves MET cycle flux and DNA methylation in CD4^+^ cells; (iii) promotes transcription of the *Ikzf2, Foxp3,* and *Ctla4* genes and downregulates transcription of the *Il17* and *Ifng* genes, restoring the Treg/Th17 cell balance; (iv) increases DNA methylation and downregulates transcription of the DRB1*1501 gene in myeloid-lineage cells. Together these alterations reduce the risk of developing MS.

The fact that the MHC class II *DRB1* locus largely determines MS genetic susceptibility, underpins the view that MS is an autoimmune disease [33]. Curiously, the strongest susceptibility gene, DRB1*1501, does not always yield an MS disease phenotype, ruling out a strict MHC class II gene-phenotype model of inheritance [34]. The incomplete penetrance of DRB1*1501 is most evident in affected MS families where the gene is identical by descent. For example, unaffected DRB1*1501-positive offspring of MS cases showed a striking over-transmission of the DRB1*1401 allele [33,35]. Similarly, among DRB1*1501-positive sibling pairs, the unaffected sibs showed over-transmission of the DRB1*01 allele while the affected sibs showed under-transmission of this allele [36]. Incomplete penetrance of the DRB1*1501 MS risk allele was attributable to inheritance of DRB1*1401 and DRB1*01 functioning as broadly acting MS resistance genes in these examples. In fact, the DRB1*14-, DRB1*01-, DRB1*11-, and DRB1*10-bearing haplotypes were all protective, but different mechanisms governed their actions [37]. The DRB5*0101 allele also demonstrated a protective effect in DRB1*1501 -positive individuals [38]. In this case, animal modeling revealed a functional epistatic interaction between DRB5*0101 and DRB1*1501 that resulted in the clonal deletion of self-reactive T cells [38]. In summary, heritable MS risk centers on the DRB1*1501 allele, but epistatic interactions with resistance alleles modify its capacity to yield an MS disease phenotype.

Studies of DRB1*1501 transmission in affected MS families have also revealed epigenetic mechanisms that govern development of the MS disease phenotype [28,39]. Distortions in transgenerational disease transmission have been observed [40]. Unexpectedly, affected aunts had a significantly lower DRB1*1501 allele frequency than their affected nieces, whereas affected uncles and nephews had identical DRB1*1501 allele frequencies [41,42]. The calculated MS risk odds ratio (OR) for DRB1*1501 was ~4 based on the aunt-niece pair transmission data but only ~2 based on sibling pair transmission data. These findings suggest interactions between resistance genes, hormones, and the environment impinge on the DRB1*1501 disease risk allele where they act by epigenetic mechanisms to determine its penetrance. As discussed below, a vitamin D responsive element (VDRE) was reported in the DRB1*1501 promoter [43]. Whether the resistance alleles or this VDRE are linked in any way to epigenetic mechanisms controlling DRB1*1501 gene expression is unknown (Figure 1D). Importantly, if epigenetic silencing mechanisms applied to DRB1*1501 were to fail, then an increase in the odds of an MS phenotype would be expected, as was observed in the aunt-niece pair transmission data.

Racial admixture studies also support the vitamin D, Treg cell, and DRB1*1501 epigenetic hypothesis of MS risk. Canadian MS patients with a Caucasian mother and an Aboriginal father had a greater F:M sex ratio than patients with an Aboriginal mother and a Caucasian father [44,45]. This maternal parent-of-origin effect was most evident in the transmission of DRB1*1501 to affected female offspring. Thus, there was a female bias in the epigenetic mechanisms associated with DRB1*1501. It will be important to uncover those gender-specific epigenetic mechanisms and determine how hormonal and environmental factors influence them.

New research is driving toward this goal. MS disease status has been linked to DNA hypomethylation at the *DRB1* locus in CD4^+^ T cells [29,46]. The CD4^+^ T cells from treatment naive RRMS patients had 37% less DNA methylation at a CpG island within the *DRB1* locus compared to healthy controls. The DRB1*1501 allele was more prevalent among RRMS cases than controls suggesting this risk allele probably accounted for the differential DNA methylation signal. Others confirmed this hypothesis by mapping 19 consecutive hypomethylated CpGs within exon 2 of the *DRB1* gene in DRB1*1501-positive MS patients compared to healthy controls [30]. As DNA methylation decreased, the DRB1*1501 expression levels increased in CD4^+^ T cells, monocytes, B cells, and CD8^+^ T cells. A novel DRB1*1501 variant with high DNA methylation at the exon 2 CpG island was discovered in the healthy control group. The high DNA methylation in this novel DRB1*1501 variant correlated with reduced DRB1*1501 expression in CD4^+^ T cells, monocytes, B cells, and CD8^+^ T cells.

These observations suggest that DNA methylation within exon 2 of the DRB1*1501 gene could function as an epigenetic switch controlling gene expression and MS risk. However, caution is warranted in extrapolating the case-control data to support the DRB1*1501 epigenome hypothesis of MS risk because the correlation between DRB1*1501 DNA hypomethylation and MS disease status might reflect a disease consequence (reverse causality). A pioneering longitudinal study of type 1 diabetes found specific DNA hypomethylation marks within the MHC class II *DRQ1* region in immune cells many years prior to development of disease [47]. Similar longitudinal studies could ascertain whether specific DNA hypomethylation marks within the DRB1*1501 gene exist in immune cells prior to development of MS. Given the rapid rise in female MS incidence (see below), addressing the question whether DRB1*1501 DNA hypomethylation functions as an epigenetic switch controlling gene expression and MS risk is an urgent research priority.

Racial admixture studies have begun to address this question. MS susceptibility has been mapped to DRB1*1501 in African Americans, but the European-derived and African-derived alleles showed distinct patterns of diversity, linkage disequilibrium, and risk [48]. The European DRB1*1501 alleles had a common amino acid sequence and conferred a 3-fold greater MS risk compared to the African DRB1*1501 alleles, whose sequences diverged in peptide-binding regions [49]. One interpretation is that the newer European DRB1*1501 allele may have arisen in a human population that migrated out of Africa and then undergone positive selection, while the older ancestral DRB1*1501 allele may have continued to undergo mutation and selection in Africa. Like family studies, longitudinal studies of racially-mixed populations could test the DRB1*1501 epigenome hypothesis of MS risk. In longitudinal studies, it will be important to use genotyping methods capable of detecting novel DRB1*1501 variants with DNA methylation differences. Parsing the data according to gender, age, and vitamin D status would also be beneficial. It is imperative to understand whether the DRB1*1501 gene epigenetic marks in the immune cells are fixed in gestation when hematopoietic progenitors form, or dynamic in post-natal life. If epigenetic marks in the immune cells are dynamic, knowledge of mechanisms that modify these epigenetic marks is needed.

### Ultraviolet Light and Vitamin D

A purely genetic etiology for MS risk has been excluded in favor of a model wherein a strong, latitude-linked environmental component interacts with a modest heritable component to determine MS risk. This environmental component is non-transmissible and acts at the population level in a female-biased manner. The evidence supporting this gene-environment interaction model has been reviewed [2,50,51]. It includes the high 60% to 75% MS disease discordance rate between monozygotic twins, the incomplete penetrance of the DRB1*1501 susceptibility genotype, the latitudinal gradient and female bias in MS prevalence, the rising MS incidence rate especially in young girls (see below), the migration- and diet-induced changes in MS risk, and the equivalent MS risk between non-biological relatives of an MS case and the general population. The gene-environment interaction model implies that many MS cases might be prevented through timely modification of environmental exposures.

The major latitude-linked component in MS risk was postulated to be low sunlight exposure [52]. MS prevalence varies ~400-fold [53], correlating inversely with a 400-fold variation in ultraviolet B (UVB) irradiance [54]. The negative correlation coefficient between UVB exposure and MS prevalence was very strong, −0.9 [52]. High ambient UVB irradiation during childhood and adolescence showed the strongest inverse correlation with MS risk. These data imply that the UVB-linked factor exerts its greatest influence on MS risk in the first two decades of life, before and/or during the prodromal period when the pathological processes that cause demyelination are beginning, but demyelinating disease is not yet clinically evident [55,56].

The vitamin D pathway is widely believed to be the major biological signal transducer for the protective effects of cutaneous UVB light exposure as regards MS risk [2]. Cutaneous exposure to high energy UVB photons (290–315 nm) generates vitamin D_3_. This photolysis reaction has functioned as a sunlight sensor and signal transducer throughout >750 million years of evolution, enabling organisms to coordinate mitochondrial energy generation, cellular metabolism and growth, cell differentiation and death, immunity and reproduction according to cues from the sunlight [57]. The ultimate UVB photon signal transducing molecules are the small lipophilic hormone, 1,25-(OH)_2_D_3_, and the VDR, a hormone-responsive transcriptional regulator [58]. There is no other known cutaneous photolysis reaction that rivals vitamin D_3_ synthesis as a UVB photon sensor and signal transducer. Vitamin D_3_ synthesis varies seasonally at high latitudes, reaching a nadir two months after the winter solstice, and a zenith two months after the summer solstice. The higher the latitude, the longer is the period of sunlight and vitamin D deprivation. Below we describe vitamin D metabolism and signaling. Relatively recent life-style changes have reduced population sunlight exposure causing a global epidemic of hypovitaminosis D (reviewed in [59]).

Genetic studies have yielded robust evidence that the vitamin D_3_ system mediates sunlight’s protective effects as regards MS risk. Rare complete loss-of-function mutations in the *CYP27B1* gene encoding the 1-alpha hydroxylase enzyme that produces 1,25-(OH_2_)D_3_ from 25-hydroxyvitamin D_3_ (25-(OH)D_3_) correlated strongly with MS risk [60–63]. In one MS-affected family pedigree going back four generations, 35 of 35 MS patients inherited a defective *CYP27B1* allele that impeded vitamin D hormone synthesis; the odds of this inheritance pattern occurring randomly are 1 in a billion [61]. This and other similar MS family studies indelibly mark the 1,25-(OH)_2_D_3_ biosynthetic enzyme as a key determinant of MS risk [62,63].

Mendelian randomization studies have exploited the genetically-determined variation in serum 25-(OH)D_3_ levels to analyze the relationship between life-long exposure to hypovitaminosis D and MS risk [64–67]. These studies utilized single nucleotide polymorphisms (SNPs) in or near *GC* (vitamin D binding protein), *DHCR7* (7-dehydrocholesterol reductase), *CYP2R1* (vitamin D-25-hydroxylase), and *CYP24A1* (1,25-dihydroxyvitamin D-24-hydroxylase) [67]. Each SNP was associated with an independent and significant negative impact on serum 25-(OH)D_3_ levels, and their effects were additive. Nearly 40,000 individuals were sorted into groups according to the number of inherited 25-(OH)D_3_-reducing SNPs. The frequency of MS cases within these groups varied inversely with the genetically-determined 25-(OH)D_3_ level. Plotting the Mendelian randomization study data, assuming a log-linear relationship, and fitting an equation revealed that the probability of developing MS was >11-fold higher in the group with severe hypovitaminosis D (25-(OH)D_3_ < 10 nmol/L) compared to the minimal MS risk group with 25-(OH)D_3_ ~111 nmol/L. The physiological 25-(OH)D_3_ level is believed to be ~115 nmol/L [68,69]. These Mendelian randomization study data indelibly mark the serum 25-(OH)D_3_ level as a key determinant of MS risk.

Longitudinal studies have demonstrated that environmentally-determined low circulating 25-(OH)D_3_ levels early in life correlated with a high risk of developing MS later in life. Insufficient maternal 25-(OH)D_3_ during pregnancy correlated with a 2-fold increased risk of MS in the offspring [70]. Furthermore, neonatal blood 25(OH)D_3_ levels also correlated with a 2-fold increased risk of MS later in life [71]. MS risk was highest among individuals in the bottom quintile, 25-(OH)D_3_ < 20.7 nmol/L, and lowest among those in the top quintile, 25-(OH)D_3_ ≥ 48.9 nmol/L; the OR for the top vs the bottom quintile was 0.53. The relationship between environmentally-determined low vitamin D status and high MS risk was stronger in women than men [72–75]. Collectively, these studies mark a genetically- or environmentally-determined low 25-(OH)D_3_ level as a key determinant of MS risk, and in addition, suggest that risk acquisition attributable to low vitamin D_3_ status begins in gestation and continues in childhood and adolescence, particularly in females. It is a concern that risk acquisition linked to low vitamin D_3_ status begins in gestation because these data point to epigenetic mechanisms that may not be reversible.

Some investigators have rejected the vitamin D-MS hypothesis writing: “If vitamin D deficiency causes MS, as smoking causes lung cancer, then we would expect this to be true across all racial/ethnic groups” and it is not true in blacks [76]. This suggested parallel is false. Smoking *acts independently* of genotype, gender, other environmental exposures, and age to determine cancer risk [77]. In contrast, vitamin D deficiency is hypothesized to *act in concert* with genotype, gender, and other environmental exposures (e.g., obesity) from birth through adolescence to determine MS risk.

A case-control study of the racial disparity in MS risk was cited as the basis for rejecting the vitamin D-MS hypothesis [78]. We examined this report carefully because the sunlight-vitamin D_3_ debate bears on efforts to reduce MS incidence [59]. Adult female and male MS patients of European ancestry, mixed African and European ancestry, and matched controls living in Southern California were enrolled. At enrollment, vitamin D supplement use, cumulative lifetime UV exposure (estimated from recalled data), and a current plasma 25-(OH)D_3_ level were recorded. Importantly, the 25-(OH)D_3_ and UV exposure MS case *vs* control data showed highly similar inverse correlations for European Americans and African Americans. The inverse correlation between 25-(OH)D_3_ and MS reached significance (*p* < 0.05) in European Americans as did the inverse UV-MS correlation in both races. However, the investigators rejected the vitamin D hypothesis in favor of the UV-MS hypothesis based on the insignificance (*p* > 0.05) of the inverse correlation between 25-(OH)D_3_ and MS in African Americans.

This two-group parallel study design had important limitations. At 34°N latitude, UVB-catalyzed vitamin D_3_ synthesis occurs year round and seasonal 25-(OH)D_3_ variation is muted, which quite likely decreased the difference between the group 25-(OH)D_3_ means. Furthermore, only the UV assessment included the developmental period when 25-(OH)D_3_ is postulated to act. Also, including vitamin D supplement users in the MS case (12%) and control (3%) groups confounded only the 25-(OH)D_3_ data. Most importantly, with 247 African American subjects, there was only a 34% likelihood of detecting a significant 25-(OH)D_3_ difference between the group means, given the differences and variation in the means. With 514 European American subjects enrolled, the likelihood of detecting a significant difference was 86%. To detect significant 25-(OH)D_3_ differences with 90% confidence, >1000 subjects would have been required. In sharp contrast, the study had 99% confidence of detecting a significant difference in the UV assessments for both races. Thus, the statistical insignificance of a single measurement in a deeply-flawed and underpowered study provided the basis for rejecting the vitamin D_3_ hypothesis and all its supporting data. Reviews have emphasized the problem of advancing unsupportable claims based on statistical insignificance [79]. These considerations lead us to recommend that this report not be considered further.

Other investigators have labeled the vitamin D-MS hypothesis “the dog’s dinner”, referring to the mistaken notion that the hypothesis predicts “treatment of MS with vitamin D supplements would help ameliorate the course of established MS” [80]. It is imperative not to conflate MS disease prevention with treatment. In animal modeling studies, vitamin D_3_ supplementation inhibited EAE induction only in females [81], but did not inhibit established EAE in females or males [82]. There was an opportune developmental window between weaning (age 3–4 weeks) and sexual maturity (age 6 weeks) for introducing and continuing vitamin D_3_ supplementation in order to observe inhibition of EAE induction [81,83,84]. There was also an opportune window after sexual maturity but before old age (age 12 weeks) for inducing EAE in order to observe vitamin D_3_-mediated disease inhibition. These windows of opportunity in the rodent correspond to data from human subjects.

A recent EAE study in marmosets echoed the rodent and human data [85]. Marmosets given a new dietary supplement that provided a significantly higher intake of vitamin D_3_ (as well as B vitamins) showed decreased EAE incidence and severity, diminished spinal cord demyelination, and reduced proinflammatory T cell responses to myelin oligodendrocyte protein relative to marmosets given the previous supplement. The role of supplementary vitamin D_3_ in the marmoset EAE outcome is uncertain because the 25-(OH)D_3_ levels were neither measured nor discussed. It is noteworthy that folic acid (vitamin B_11_) and cobalamin (vitamin B_12_) have known roles as substrate and cofactor, respectively, for MET cycle enzymes that provide single carbon units for DNA methylation (see below).

Two expert panels have examined the evidence for and against the vitamin D_3_-MS hypothesis and concluded: (i) hypovitaminosis D has a causal role in MS risk particularly in women, (ii) the global incidence of MS in women is rising rapidly, and (iii) the global downward trend in population vitamin D status may be contributing to the global upward trend in female MS risk [59,86]. Whether vitamin D_3_ status influences the epigenetic mechanisms controlling expression of the DRB1*1501 gene or any other gene relevant to MS risk is unknown. In summary, it is reasonable to suggest that vitamin D is an environmental factor with great influence on MS development, given a disease-susceptible genotype; a window of opportunity appears to exist between gestation and adolescence for intervening to lessen the MS risk. Scientists investigating how and in whom to test interventions intended to restore circulating 25-(OH)D_3_ to physiological levels [68] face a daunting complexity of genetic, epigenetic, hormonal, nutritional, geographic, and cultural influences that must be considered [87].

### Obesity and Vitamin D Deficiency

Female adolescent obesity has been firmly linked to an increased risk of MS [88]. Post-pubertal adolescents who were overweight (BMI ≥ 30 kg/m^2^) before age 20 had a roughly 2-fold increased risk of MS compared to adolescents of normal weight [89–93]. This association was especially apparent in extremely obese girls, where the odds of developing MS were nearly 4-fold greater compared to girls of normal weight [94]. Importantly, girls who were both obese and DRB1*1501 positive had a 16-fold increased risk of MS compared to non-obese girls without this genetic risk factor, highlighting a positive interaction between obesity and DRB1*1501 [95]. These data are consistent with a Mendelian randomization study wherein each 1 S.D. increase in genetically-determined BMI increased the odds of developing MS by 41% [96]. Thus, it appears that obesity, especially in combination with the DRB1*1501, is causally associated with female MS risk.

Given the correlation between female adolescent obesity and MS risk and the Mendelian randomization study data supporting a causal relationship, the question arises would MS patients generally be more obese than the population at large. Obesity is only one factor in a complex cluster of interacting disease-promoting and disease-inhibiting factors. Consequently, not all MS patients would be expected to be obese. The OR of developing MS can be envisioned as the sum of many factors in a complex risk equation, where each factor carries some weight according to its independent contribution to the overall OR. Without attempting to specify the OR for all independent and interdependent factors, one can imagine that the neurodegenerative and autoimmune disease processes characteristic of MS will begin when the sum of these terms reaches some risk threshold. For example, a young female-positive Scandinavian of average BMI who develops symptomatic mononucleosis would have a high OR of developing MS although she is not obese.

Although obesity is neither necessary nor sufficient to cause MS, it is still of interest to ask if MS patients would have a higher average BMI than the general population. An intriguing recent report addressed this question by evaluating longitudinal changes in BMI after MS onset, and the relationship between BMI and MS severity in a cohort of adult-onset MS patients and matched healthy controls [97]. The baseline BMI in the MS patients, mean age ~44 years, was significantly higher than the age- and sex-matched controls (mean difference = 0.57; *p* = 0.008). However, the BMI of controls increased 0.069 kg/m^2^ per year with advancing age, whereas the BMI of MS patients increased only 0.035 kg/m^2^ per year. This trend would result in lower average BMI for older MS patients compared to controls, which has been documented in patients with longstanding MS [89]. There was also a remarkable sex-specific association between BMI changes and MS disability in the longitudinal study [97]. Each one unit increase in BMI correlated with a 0.033 unit increase in MS disability in women, but a 0.053 unit decrease in MS disability in men. There is presently no biological explanation for the female-specific correlation between increasing BMI and MS disability.

Whether obesity and vitamin D deficiency are independent or interdependent MS risk factors is unclear. We suggest they are interdependent risk factors. In obese individuals, fat-soluble 25-(OH)D_3_ partitions into adipose tissue reducing its concentration in the circulation [98]. Basal circulating 25-(OH)D_3_ levels were lower in obese subjects (BMI ≥ 30 kg/m^2^) than in lean subjects (BMI < 25 kg/m^2^). Importantly, obese and control subjects produced equivalent amounts of vitamin D_3_ in response to cutaneous UVB irradiation, but the increase in circulating 25-(OH)D_3_ was 57% lower in the obese subjects than in lean subjects. Similar findings applied to ingestion of vitamin D3. Further, a Mendelian randomization study revealed that each 10% higher genetically-determined BMI score correlated with a 4.2% decrease in circulating 25-(OH)D_3_ [99]. Together, the data demonstrate that obesity reduces circulating 25-(OH)D_3_.

Emerging data on the satiety hormone, leptin, points to another cause-effect relationship between obesity and vitamin D insufficiency. Adipocytes produce leptin which travels to the brain, binds to leptin receptors (LepR) on hypothalamic neurons, and signals a reduction in appetite [100]. Immune cells also express the LepR. In monocytes and dendritic cells, LepR signaling upregulates cell activation, migratory capacity, phagocytosis, antigen presentation, and pro-inflammatory cytokine production. In CD4^+^ T cells, LepR signaling promotes Th1 and Th17 cell proliferation, differentiation, and cytokine production, while inhibiting Treg cell development and function. Thus, leptin promotes protective inflammatory responses when energy stores are sufficient, but when energy stores are low, as they are during periods of famine, decreased leptin production removes this stimulus to preserve energy for essential organ function [101]. In obese individuals, excess leptin production promotes the pro-inflammatory immune phenotype.

Recently, the relationship between obesity, vitamin D, and leptin was investigated [102]. A one-year lifestyle intervention in obese subjects decreased visceral adipose tissue volume 26%, decreased plasma leptin by 27%, and increased plasma 25-(OH)D_3_ by 27%. Importantly, leptin correlated inversely with plasma 25-(OH)D_3_ even after adjusting for changes in adiposity. Leptin-deficient mice overexpressed the *Cyp27b1* gene and had elevated 1,25-(OH)_2_D_3_ levels [103]. Injecting leptin reduced *Cyp27b1* gene expression and 1,25-(OH)_2_D_3_ levels. Other research demonstrated that 1,25-(OH)_2_D_3_ signaling through three VDREs upstream of the mouse *lep* gene reduced leptin mRNA production by 84% in mouse adipocyte cultures [104]. These data show a mutually antagonistic relationship between leptin, the satiety-sensing hormone, and 1,25-(OH)_2_D_3_, the sun-sensing hormone. In summary, it is reasonable to hypothesize that obesity and vitamin D deficiency are interdependent MS risk factors, given biochemical data demonstrating vitamin D metabolite partitioning into adipose tissue and antagonism between leptin and 1,25-(OH)_2_D_3_; a window of opportunity appears to exist to lessen the MS risk in girls and reduce the accumulation of MS disability in women by resolving the dual epidemics of obesity and vitamin D deficiency.

## MYELIN

### Oligodendrocytes and Myelination

The acquisition of myelin in vertebrate evolution allows very fast information processing in the small space of the vertebrate brain [105]. The myelin sheath insulates and provides trophic support to the axon between the non-myelinated nodes of Ranvier. This structural arrangement restricts action potentials to the exposed nodes, accelerating nerve signal transmission up to 100-fold as the action potential jumps from one node to the next (see Figure 2 in [106]). In the vertebrate CNS, axonal action potential firing induces myelination. Myelinating oligodendrocytes extend membrane processes to form spiral, multilamellar myelin sheaths surrounding axons between the nodes of Ranvier. In humans, myelination begins in late gestation, and increases rapidly in the postnatal period of rapid body growth and axon elongation. During this period, an oligodendrocyte grows its myelin membranes by an astonishing −5000 μm^2^/day. Mechanisms that stimulate myelin thickening at the innermost membrane and thin the myelin sheath nondestructively at the outermost membrane provide opportunities for remodeling, plasticity, and repair throughout life [106].

Brain-derived neurotrophic factor (BDNF) supports myelination through its effects on oligodendrocytes [107]. Mice with oligodendrocyte-specific disruption of the *Trkb* gene encoding the BDNF receptor had normal numbers of mature oligodendrocytes and myelinated axons, but the thickness of the myelin sheath was significantly reduced. Thus, BDNF-TrkB signaling promoted myelin wrapping of axons but played no role in oligodendrocyte maturation or initial membrane contacts between oligodendrocytes and axons. Recent research has demonstrated that mature oligodendrocytes also participate in remyelination [108].

### Myelin Structure

The multilamellar myelin sheath is comprised of lipids and proteins. Viewed in cross-section, the myelin sheath is composed of oligodendrocyte (i) extracellular membrane, (ii) cytoplasmic membrane, (iii) a thin protein layer, (iv) opposing cytoplasmic membrane, and (v) opposing extracellular membrane, organized in a multilayered stack of up to 160 membrane layers spiraled around the axon (see Figure 2 in [109]). The major dense tracts observed in transverse electron micrographs represent the condensed cytoplasmic myelin membranes, whereas the tightly opposed extracellular membranes are observed as lighter regions between the dense tracts. Newer imaging techniques have revealed a cytoplasmic channel network within the myelin membrane layers that facilitates distribution of cytoplasmic molecules and nutrients.

### Myelin Proteins

Myelin proteolipid protein (PLP) constitutes >50% by weight of the protein embedded in the myelin sheath [110]. PLP is critically important to myelin biosynthesis, structure, and function, as evidenced by the very high degree of *PLP1* gene sequence conservation (human and mouse genes are identical) and the severity of the X-linked demyelinating diseases observed in *PLP1* mutant individuals [111]. Targeting the *Pip* gene in adult mice reduced the abundance of PLP by about 50% as the myelin slowly turned over [112]. Importantly, this reduction caused the entire spectrum of neuropathological changes previously associated with a developmental lack of PLP. The PLP structure is noteworthy for the four helical regions that span the oligodendrocyte plasma membrane, and the six long-chain FA (mainly palmitic acid with some oleic and stearic acids) in thioester linkage with cysteines at the intracellular face. These structures and post-translational modifications give PLP it’s highly hydrophobic character and natural affinity for cholesterol and phospholipids. These affinities allow PLP to enrich cholesterol by molecular association, and cotransport it as membrane microdomains coalesce to form nascent myelin in the oligodendroglial secretory pathway [113].

Myelin basic protein (MBP) is the second most abundant protein in myelin, comprising 30% by weight of the myelin sheath proteins [110]. This protein is rate-limiting for myelination. It is responsible for the compaction of the multilamellar membrane structure which is the signature feature of mature myelin. MBP acts as an electrostatic glue, its positively-charged arginine and lysine residues forming strongly adhesive charge-charge interactions with the negatively-charged phospholipids on the inner leaflets of the opposing membrane bilayers. As MBP brings the inner leaflets into close proximity in a membrane zippering process, the water is excluded and the membrane becomes compact and stable [114]. Changes in composition that affect the electrostatic or hydrophobic interactions in the myelin sheath lead to swelling, instability, and demyelination.

Other myelin proteins include claudin-11 (7% by weight), cyclic-nucleotide phosphodiesterase (2–4% by weight), myelin-associated glycoprotein (1% by weight), and myelin oligodendrocyte glycoprotein (0.05% by weight). Claudin-11 is noteworthy among these minor proteins. It is a tight junction protein that associates with a K^+^ channel (Kv3.1) to regulate oligodendrocyte proliferation, migration, and myelin production [115].

### Myelin Lipids: Cholesterol, Sphingomyelin, and Nervonic Acid

The CNS is a lipid rich tissue. The myelin sheath harbors nearly half of the brain’s lipids. Myelin itself is about 75–80% lipid by dry weight, with cholesterol, phosphatidylcholine, sphingomyelin, ceramide, glucosyl-ceramide, and sulfatide being the major lipid species [109]. Lipoprotein particles do not cross the blood-brain barrier (BBB) under normal circumstances, so the lipids needed for myelin synthesis must be produced de novo in the CNS. Studies in monkeys have established that myelin remodeling and repair occur throughout life [116], necessitating continuous de novo lipid synthesis in the CNS. The need for de novo lipid synthesis is greater in demyelinating diseases like MS [117].

Cholesterol is an essential component of every vertebrate cell and organelle membrane. Remarkably, myelin membranes harbor 80% of total brain cholesterol, representing the largest pool of free cholesterol in the human body [105,118,119]. In the oligodendrocyte myelin membrane, >25% of the lipid dry weight is cholesterol, about 2-fold more than plasma membranes of neurons, astrocytes, and microglial cells. Cholesterol availability is rate-limiting for myelination in the CNS [118]. Lipid biosynthesis is controlled by the mammalian target of rapamycin signaling pathway, specifically mTORC1, sterol regulatory element binding protein (SREBP), and the SREBP cleavage-activating protein [120]. Upon activation, SREBP initiates transcription of the *HMGCR* gene encoding 3-hydroxy-3-methylglutaryl-coenzyme A reductase (HMG-CoAR), the rate-limiting enzyme in cholesterol biosynthesis [118]. Cholesterol synthesis is performed mainly by neurons during embryogenesis, by oligodendrocytes during postnatal myelination, and by astrocytes during myelin repair and replacement in adult life.

Several clinical trials have investigated statin inhibitors of HMG-CoAR and cholesterol biosynthesis as add-on therapy for MS patients [121]. The rationale underlying these trials was that statins would reduce the cholesterol biosynthetic intermediates that drive RORγt-dependent CD4^+^ Th17 cell differentiation [22,122]. Unfortunately, this reasoning did not consider the requirement for de novo cholesterol biosynthesis in the CNS to support myelin biosynthesis, and the compromised BBB observed in MS patients [116,118]. The clinical trial results showed a higher proportion of MS patients in relapse, new T2 lesions, and greater whole brain atrophy in the statin groups compared to the non-statin groups. These findings reinforce the conclusion that MS patients have a greater need for de novo cholesterol biosynthesis in the CNS than healthy controls [117]. We suggest that the ongoing debate about future statin clinical trials in MS patients consider this need for de novo cholesterol biosynthesis.

**Figure 2.**
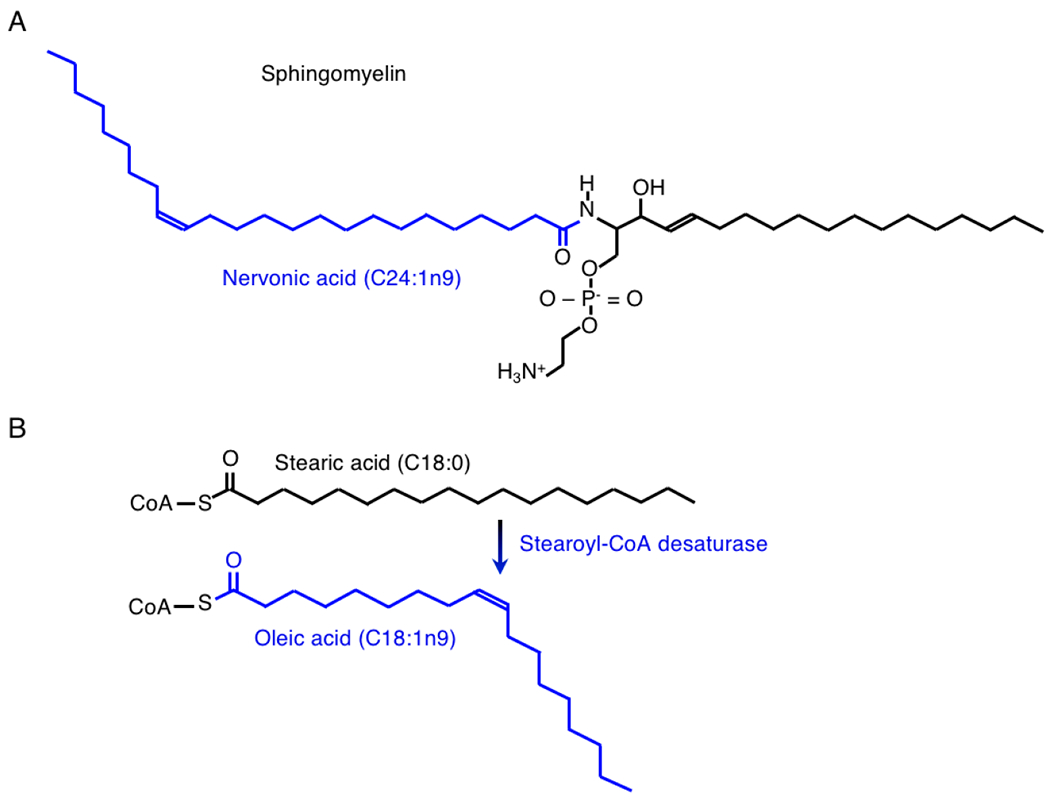

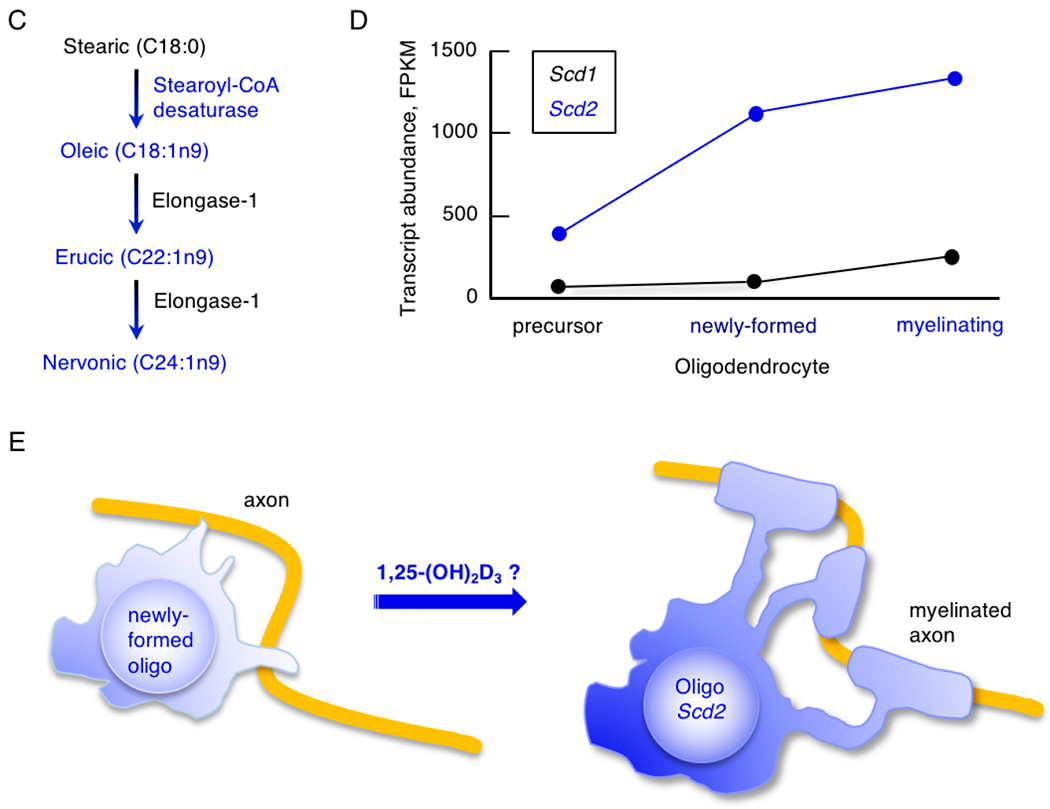
The vitamin D-stearoyl-CoA desaturase hypothesis of MS risk. (**A**) Sphingomyelin with nervonic acid (C24:1n9) in amide linkage is a predominant constituent of the myelin sheath; sphingomyelin and nervonic acid were sharply decreased in MS myelin. (**B**) Stearoyl-CoA desaturase converts saturated stearic acid (C18:0) to mono-unsaturated oleic acid (C18:1n9), which is the rate-limiting step in nervonic acid biosynthesis. (**C**) Elongase-1 converts oleic acid into erucic acid (C22:1n9) and then nervonic acid by addition of two carbon units. (**D**) The murine *Scd1* and *Scd2* transcript levels increase as oligodendrocyte precursor cells develop into mature myelinating oligodendrocytes, with *Scd2* transcripts being 5.4-fold more abundant than *Scd1* transcripts in myelinating oligodendrocytes. FPKM, fragments per kilobase of transcript per million mapped reads. The *Scd* transcript graphs are based on data from a brain single cell transcriptome analysis (http://www.brainrnaseq.org) [127]. (**E**) Hypothesized positive role of 1,25-(OH)_2_D_3_-VDR-signaling in oligodendrocyte maturation, *Scd2* gene expression, nervonic acid (24:1n9) biosynthesis, myelin stability and myelin repair. Blue objects and text represent proposed protective actions of the vitamin D system.

Sphingomyelin is a second predominant constituent of the myelin sheath (Figure 2A) [123]. Sphingomyelin is a precursor of bioactive lipid signaling molecules. Sphingomyelin increases from 2% of brain lipids at birth to 15% at 3 years such that the ratio of sphingomyelin to ceramide is 9:1 in the adult [124]. In hereditary Niemann-Pick disease, a defect in sphingomyelinase causes sphingomyelin to accumulate in spleen, liver, lungs, bone marrow, and brain, where it causes irreversible neurological damage.

Fatty acids (FA) are important components of sphingomyelin, phosphatidylcholine, ceramide, glucosyl-ceramide, and sulfatide [123]. The CNS expresses genes for FA synthesis and is autonomous in lipid metabolism [125]. Targeting FA synthase in rodent oligodendrocyte precursor cells had no impact on oligodendrocyte differentiation, but myelin lipid composition was altered, stability was reduced, and remyelination was poor after toxin-induced demyelination [126]. Dietary lipids only partially compensated for oligodendrocyte-specific FA synthase deficiency. Thus, FA synthase was essential for oligodendrocyte myelin synthesis in the postnatal period and after myelin injury in the adult.

In normal brain development, astrocytes contribute a substantial fraction of the lipids incorporated into myelin. Astrocytes produced ApoE and released cholesterol to LDL particles for uptake by oligodendrocytes and incorporation into myelin [128,129]. Disruption of lipid metabolism by embryonic deletion of SREBP cleavage-activating protein in astrocytes alone or together with oligodendrocytes caused persistent hypomyelination [130]. Importantly, a lipid-enriched diet restored a myelinating phenotype in these conditional mouse mutants. These data show that astrocyte lipid synthesis and horizontal lipid flux to oligodendrocytes for myelin synthesis and repair is a major feature of normal brain development. They also show that over time, dietary lipids can compensate for diminished lipid synthesis in the brain.

Nervonic acid (C24:ln-9), a mono-unsaturated FA (MUFA), is the major FA component of the sphingomyelin, sulphatides, and cerebrosides in the human brain (Figure 2A). During early human development, the nervonic acid content of sphingomyelin increased 6-fold from −300 nmol/g at age 1 year to >1900 nmol/g at age 6 years [131]. In adult white matter, nervonic acid, at 36%, was also the major FA component of sphingomyelin [132,133].

Stearoyl-CoA (Δ9) desaturase (SCD) carries out the rate limiting step in nervonic acid (C24:ln9) biosynthesis (Figure 2B) [134]. This enzyme converts saturated palmitic acid (C16:0) or stearic acid (C18:0) into their mono-unsaturated counterparts palmitoleic acid (C16:ln7) and oleic acid (C18:ln9), respectively. Oleic acid is a major FA component of myelin, and a neurotrophic factor in the developing brain, where it promotes neural cell differentiation and axonal growth [135,136]. Subsequently, FA elongase-1 lengthens oleic acid (C18:1n9) into gadoleic acid (C20:1n11), erucic acid (C22:1n9), and finally, nervonic acid (C24:1n9) (Figure 2C) [137].

Human and rodent oligodendrocytes express *SCD* transcripts (http://www.brainrnaseq.org) [127]. The murine *Scdl* and *Scd2* transcripts increased ~8.4-fold and ~3.5-fold, respectively, as oligodendrocyte precursor cells (OPC) developed into newly-formed oligodendrocytes (Figure 2D). In the mature myelinating oligodendrocytes, *Scd2* transcripts were 5.4-fold more abundant than *Scdl* transcripts. In mature astrocytes, *Scd2* transcripts were 9-fold more abundant than *Scdl* transcripts, and similar to *Scd2* transcript abundance in newly-formed oligodendrocytes. The *Scdl* transcripts were expressed in astrocytes of the cortex, hippocampus, and striatum throughout the mouse lifespan. The human homolog of the mouse *Scd2* gene, *SCD*, was also expressed at high levels in mature myelinating oligodendrocytes and astrocytes, with expression being 2.6-fold higher in the oligodendrocytes. These data suggest that SCD is a critically important enzyme to supply nervonic acid (C24:1n9) for myelin biosynthesis in the CNS. It is especially significant that *SCD* transcripts were markedly decreased in MS lesion samples obtained at autopsy compared to healthy control samples (Figure 3 in [138]).

### Myelin Lipids in MS and EAE

Striking myelin lipid changes have been reported in MS patients. The sphingomyelin, cerebroside, and sulfatide fractions from MS myelin had substantially more palmitic (C16:0) and stearic (C18:0) acids and less nervonic acid (C24:1n9) than control myelin (Table 1) [133]. Sharply decreased sphingomyelin and increased phospholipids were found in MS myelin [139]. In MS white matter, C16:0-and C18:0-ceramide elevation was associated with oligodendrocyte apoptosis [140]. Whether the altered myelin lipid content was a cause or consequence of MS is unknown.

Myelin sphingolipids from rodents with EAE showed similar alterations (Table 2) [141]. There were no changes in cholesterol, hydroxylated or non-hydroxylated cerebroside, or phosphatidylethanolamine. However, cerebroside sulfatide was lower and phosphatidylserine was higher within the extracellular face of the myelin membrane in EAE samples compared to controls. Furthermore, phosphatidylcholine and sphingomyelin were 26% and 67% lower, respectively within the extracellular and cytoplasmic faces of the EAE myelin membrane than controls.

Analysis of the FA in myelin lipids revealed that nervonic acid (C24:1n9) comprised nearly 2% of FA in the total brain lipid fraction from control samples, but it was completely undetectable in samples from rodents with EAE (Table 3) [137]. Although stearic acid (C18:0) and oleic acid (C18:1n9) were not significantly different comparing EAE samples to control samples, arachidonic acid (C20:4n6) was increased more than 3-fold in the EAE samples. This increase suggests a shift to the polyunsaturated FA synthesis pathway beginning with linoleic acid (C18:2n6) and proceeding to arachidonic acid (C20:4n6). Once released from membrane phospholipids, arachidonic acid (C20:4n6) is metabolized via cyclooxygenase into prostaglandins or via lipoxygenase into lipoxins to generate pro-inflammatory eicosanoids [142].

In summary, myelin from MS patients and EAE rodents showed sharply decreased levels of sphingomyelin and nervonic acid (C24:ln9) compared to healthy control samples. Investigators have hypothesized that nervonic acid (C24:ln9) biosynthesis is silenced and lipid metabolism is shifted into pro-inflammatory arachidonic acid production during acute CNS inflammation [133,137]. The mechanism that silences nervonic acid (C24:ln9) biosynthesis in the inflamed CNS is unknown. Two possibilities are downregulation of SCD or FA elongase in oligodendrocytes and/or astrocytes by inflammatory mediator signaling. Either of these would deplete the supply of nervonic acid (C24:ln9) for sphingolipid synthesis.

Binding of TNF-α to TNF-receptor-1 activates sphingomyelinase, which hydrolyzes sphingomyelin to ceramide and phosphorylcholine [143]. Inhibiting TNF-α production prevented the oligodendrocyte apoptosis and demyelination that is normally observed in EAE disease [144], suggesting sphingomyelinase activation could be the cause of reduced sphingomyelin in EAE. The actions of TNF-α may explain why C16:0- and C18:0-ceramides were elevated in MS white matter and were associated with oligodendrocyte apoptosis [140]. Ceramide is a potent pro-apoptotic lipid second messenger in oligodendrocytes [145]. Putting these observations together, it is conceivable that the sequential actions of TNF-a, sphingomyelinase, and ceramide could deplete sphingomyelin and nervonic acid, destabilize the myelin sheath through altered electrostatic and hydrophobic interactions, and finally, kill oligodendrocytes through ceramide pro-apoptotic signaling.

A recent ground-breaking study reported that vitamin D deficiency downregulated SCD activity in the rat [146]. Female rat pups were maintained on control or vitamin D-deficient diets beginning at weaning (age 3 weeks) and continuing through adulthood and pregnancy to day 20 gestation (about age 10 weeks). The diets did not differ in FA composition. At the study’s end, serum 25-(OH)D_3_ was 9.2 ± 3.0 ng/mL in vitamin D-deficient animals and 44.7 ± 5.2 ng/mL in controls. Compared to the controls, the plasma of the vitamin D-deficient dams had 28% reduced MUFAs, increased stearic acid (C18:0), decreased oleic acid (C18:1n9), and a 45% reduced C18:0 to C18:1n9 ratio indicative of reduced SCD activity. This study is the first to document reduced SCD activity in the periphery of vitamin D-deficient rodents.

### Biophysical Properties of Myelin Membranes

Myelin instability, degradation, and ultimately an autoimmune response to denatured myelin basic protein fragments are likely consequences of sharply decreasing sphingomyelin and nervonic acid (C24:1n9) and increasing phospholipids, because these changes would increase the repulsive forces between the myelin bilayers [137,139]. The effects of replacing nervonic acid (C24:1n9) with lignoceric acid (C24:0) on the biophysical properties of myelin were investigated using large unilamellar vesicles as model membranes [147,148]. The vesicles formed by mixing cholesterol, sphingomyelins with distinct FA compositions, and lipid dyes were assessed for phase separation, intrinsic curvature, and biophysical properties. Model membranes with palmitic acid (C16:0) or lignoceric acid (C24:0) in the sphingomyelin moiety showed lateral segregation of the lipids into distinct phases. Remarkably, adding a limited amount of sphingomyelin with nervonic acid (C24:1n9) to these vesicles completely prevented lipid phase segregation. Analysis of natural biological membranes confirmed these conclusions. Nervonic acid (C24:1n9), by virtue of the shape imparted by its single ds-configured double bond, has a unique ability to pack tightly with cholesterol. The Van der Waals forces and hydrogen bonds generated by interactions between the hydrocarbon chains from adjacent lipid molecules contribute to the stability of the tightly-packed myelin membrane and to the exclusion of water [114].

To summarize, emerging data show that nervonic acid (C24:1n-9), which is the major FA component of the sphingomyelin, sulphatides, and cerebrosides in the human brain, was sharply decreased in the myelin from MS patients and EAE rodents (Figure 2). This MUFA was essential to prevent lipid phase segregation and instability in model myelin membranes. The data also show that the SCD enzymes, which catalyze the rate-limiting desaturation step in nervonic acid (C24:1n-9) biosynthesis, are highly expressed in human and rodent myelinating oligodendrocytes and mature astrocytes. Finally, very new data show that vitamin D deficiency depressed SCD activity in rat peripheral tissues. Based on these findings, we hypothesize that vitamin D deficiency similarly depresses SCD activity in myelinating oligodendrocytes and in astrocytes, and further, that loss of SCD activity in the CNS contributes to depletion of nervonic acid (24:1n9), myelin instability, demyelination, and remyelination failure. We further hypothesize that 1,25-(OH)_2_D_3_-VDR-signaling would play a positive role in oligodendrocyte maturation, *Scd* gene expression, nervonic acid (24:1n9) biosynthesis, myelin stability and myelin repair (Figure 2E).

## VITAMIN D SIGNALING IN THE IMMUNE SYSTEM

### Paracrine 1,25-(OH)_2_D_3_ Signaling to CD4^+^ T Cells

The ultimate signaling molecules in the pathway linking sunlight to its vitamin D_3_-mediated effects are the lipophilic hormone 1,25-(OH)_2_D_3_ and the VDR hormone-responsive transcriptional regulator. Knowledge of the hydroxylation enzymes that convert vitamin D_3_ into 25-(OH)D_3_ and then 1,25-(OH)_2_D_3_ has been reviewed (see Figure 1 in [149]). The mechanisms by which the 1,25-(OH)_2_D_3_-VDR complex recruits co-activators and corepressors to modify chromatin accessibility in regions with a VDRE to regulate gene transcription have also been reviewed (see Figure 1 in [150]). Here, we focus on vitamin D metabolism and signaling in immune cells.

Forty years ago, two groups reported that activated human CD4^+^ T cells express the VDR [151,152]. These reports prompted research to discover how activated CD4^+^ T cells respond to 1,25-(OH)_2_D_3_, and whether CD4^+^ T cells acquire 1,25-(OH)_2_D_3_ from the kidney by an endocrine mechanism or from cells in lymphoid tissues and inflamed sites by a paracrine mechanism. In the case of MS, indirect evidence supported paracrine signaling. Circulating 1,25-(OH)_2_D_3_ levels did not fluctuate, but MS disease activity fluctuated seasonally, correlating inversely with fluctuating 25-(OH)D_3_ levels [153–157]. At any given time, only ~2% of CD4^+^ T cells circulated in the blood, whereas 98% resided in lymphoid tissues and sites of inflammation. Finally, CD4^+^ T cells lost motility when they underwent activation to express the VDR. Collectively, these observations suggested that CD4^+^ T cells acquired 1,25-(OH)_2_D_3_ in lymphoid tissues and sites of inflammation.

Consistent with the paracrine signaling hypothesis, investigators documented 1,25-(OH)_2_D_3_ synthesis by activated, tissue-resident macrophages in human sarcoidosis, and *CYP27B1* gene expression in many non-renal tissues [158]. In fact, tissues with barrier (skin, lung, colon) or reproductive function (maternal decidua, fetal trophoblast, testis) expressed more *CYP27B1* transcripts than the kidney [159]. Furthermore, 25-(OH)D_3_ altered the human CD4^+^ T cell responses in vitro only when activated dendritic cells were present to produce 1,25-(OH)_2_D_3_ [160]. Dendritic cells and other myeloid lineage cells required activation to produce 1,25-(OH)_2_D_3_. Stimulation through pathogen-associated pattern recognition receptors (e.g., the Toll-like receptors), and/or cytokines like IFN-γ, IF-2, and IF-15 provided the activation signals [158]. Cells from the skin [159,161], lung [162], colon [163,164], brain [165], and reproductive tissues [160,166,167] all produced 1,25-(OH)_2_D_3_ in vitro. In summary, considerable evidence pointed to paracrine 1,25-(OH)_2_D_3_ signaling between activated myeloid lineage cells and activated CD4^+^ T cells, but direct in vivo evidence was lacking.

We tested the paracrine 1,25-(OH)_2_D_3_ signaling hypothesis in vivo using the EAE model. In EAE, myelin antigen-primed, CD4^+^ T cells penetrate the perivascular space, become re-activated, invade the brain parenchyma, and initiate autoimmune-mediated damage. Highly localized 1,25-(OH)_2_D_3_ synthesis in the CNS correlated with inhibition of EAE induction in vitamin D3-supplemented females [81]. We and others have also reported that 1,25-(OH)_2_D_3_ treatment inhibited EAE induction and reversed established EAE disease (reviewed in [24]).

We investigated whether direct actions of 1,25-(OH)_2_D_3_ in pathogenic CD4^+^ T cells inhibited autoimmune disease [168]. Administering 1,25-(OH)_2_D_3_ to female and male WT mice continuously beginning 3 days before immunization inhibited the cumulative EAE disease severity score by 87% relative to untreated controls. However, this protocol provided no benefits in mice with global *Vdr* gene disruption. We constructed bone marrow chimeric mice with *Vdr* gene disruption only in radio-resistant, non-hematopoietic cells, or radio-sensitive hematopoietic cells. The 1,25-(OH)_2_D_3_ inhibited the cumulative EAE disease severity score only when hematopoietic cells had a functional *Vdr* gene. In chimeric mice whose hematopoietic cells lacked a functional *Vdr* gene, the EAE disease was so aggressive that euthanasia was required. Finally, we constructed mice with CD4^+^ T cell-selective *Vdr* gene disruption. The 1,25-(OH)_2_D_3_ inhibited the cumulative EAE disease severity score by 63% in WT mice, but not in mice with CD4^+^ T cell-selective *Vdr* gene disruption. These results demonstrate direct actions of 1,25-(OH)_2_D_3_ in pathogenic CD4^+^ T cells to inhibit EAE disease induction.

### Vitamin D Regulation of CD4^+^ T Cells

Working back and forth between animal models and human disease has allowed rapid forward progress to be made in understanding how 1,25-(OH)_2_D_3_ influences VDR-expressing CD4^+^ T cells [2,24,51]. Here we summarize knowledge of 1,25-(OH)_2_D_3_ actions in CD4^+^ T cells. There are important differences between the effects of administering 1,25-(OH)_2_D_3_ to humans or animals with established demyelinating disease (treatment studies) and the effects of dietary vitamin D_3_ supplementation on susceptibility to demyelinating disease (prevention studies). Consequently, treatment and prevention study results are summarized separately.

First, we consider the treatment study results. In rodents with established EAE, dietary vitamin D_3_ supplementation provided no benefit (Figure 1 in [82]). However, in EAE diseased rodents, we found that administering a short course of 1,25-(OH)_2_D_3_, or just a single 1,25-(OH)_2_D_3_ dose plus supplementary vitamin D3, rapidly restored ambulation. Clinical recovery correlated with these changes in the CNS: (i) a doubling in the number of CD4^+^Helios^+^FoxP3^+^ Treg cells, (ii) an increase in IL-10 production, (iii) clearance of encephalitogenic CD4^+^ T cells, (iv) a loss of IFN-γ and IL-17 production, (v) reduced histological evidence of spinal cord and optic nerve pathology (reviewed in [24]). To our knowledge, 1,25-(OH)_2_D_3_ has been used to treat MS patients with encouraging results in just one pilot study, but mechanisms were not explored [169]. The 1,25(OH)_2_D_3_-regulated CD4^+^ T cell genes are summarized in Table 4. Among others, the human and murine *Vdr, Foxp3, Ikzf2, Ctla4, and Il10* transcripts increased and the *1117, 1122*, and *Iftig* transcripts decreased. Additional reported mechanisms include sensitization of encephalitogenic CD4^+^ T lymphocytes to apoptotic signals, halting of further inflammatory T cell recruitment, and enhanced expression of transcripts associated with survival of neurons and oligodendrocytes.

We recently investigated which of these mechanisms reflected rapid, primary gene expression changes attributable to 1,25(OH)_2_D_3_-VDR signaling in myelin-specific CD4^+^ T cells [32]. A single 1,25(OH)_2_D_3_ dose was administered to mice with EAE as described above [82]. This treatment rapidly induced disease remissions in WT but not T-Vdr^0^ mice with CD4^+^ T cell-specific *Vdr* gene targeting. It also halted EAE progression and induced EAE remissions in TCR-tg mice whose T cells were genetically engineered to express a myelin peptide-specific TCR. These TCR-tg CD4^+^ T cells expressed abundant *Vdr* transcripts and they increased the *Cyp24al* transcripts dramatically in response to 1,25(OH)_2_D_3_. These data indicated that 1,25(OH)_2_D_3_ induced EAE disease remissions by acting directly on activated, myelin peptide-specific CD4^+^ T cells in a VDR-dependent manner. To evaluate very early protein expression changes we collected TCR-tg CD4^+^ T cells 7 h post EAE treatment and submitted the extracted proteins for tandem mass spectrometry analysis.

Fourteen of >3000 unique proteins identified and quantified showed significant differential expression comparing samples from 1,25(OH)_2_D_3_-treated and placebo-treated animals [32]. We searched for proteins related to TCR signaling, apoptosis, and cytokines, but none of these were among the 14 differentially expressed proteins, suggesting these may be secondary gene expression changes. However, among rapidly upregulated proteins of known function, the largest increase was BHMT1 (13-fold, *p =* 3.3 × 10^−5^; <10% FDR). Helios (also known as IKAROS family zinc finger 2 protein) was a second protein of high interest. Helios enhances *Foxp3* gene expression in CD4^+^ Treg cells [200–202]. Importantly, impaired expression of Helios resulted in an unstable CD4^+^ Treg phenotype, defective CD4^+^ Treg activity and autoimmunity in mice [16].

The MET cycle uses transmethylation enzymes to recycle toxic HCY into MET (Figure 1B) [203]. There are two HCY transmethylation enzymes; BHMT1 uses Zn^2+^ ion as a cofactor and betaine as the methyl donor, whereas methyltetrahydrofolate:homocysteine methyltransferase (MTR) uses vitamin B_12_ (cobalamin) as a co-factor and 5-methyltetrahydrofolate as the methyl donor. Confirming the proteomics data, we observed 1,25(OH)_2_D_3_-mediated upregulation of *Bhmtl* transcripts and BHMT1 enzyme activity in a VDR-dependent manner in the CD4^+^ T cells. We did not observe *Mtr* transcripts in the CD4^+^ T cells. Demonstrating the importance of 1,25(OH)_2_D_3_-VDR signaling for BHMT1 enzyme activity in CD4^+^ T cells, we found that mice with CD4^+^ T cell-specific *Vdr* gene targeting developed hyper-homocysteinemia (HHcy) when they were immunized to induce EAE. HHcy did not develop in WT mice. This observation indicates that the activated CD4^+^ T cells released HCY into the circulation when they could not recycle it.

MET as S-adenosylmethionine (SAM) is the major methyl group donor for all methylation reactions. Methyl group transfer from SAM to a methyl acceptor like DNA or protein generates *S*-adenosylhomocysteine (SAH). If SAH is not hydrolyzed to adenosine and HCY and the HCY removed, SAH accumulates and inhibits all methylation reactions. Consistent with the in vivo BHMT1 data, we found that adding 1,25(OH)_2_D_3_ to activated WT CD4^+^ T cells in vitro increased global DNA methylation. This change did not occur when the CD4^+^ T cells lacked a functional *Vdr* gene. Regarding Helios, we found that 1,25(OH)_2_D_3_ also rapidly enhanced *Ikzf2* transcripts and Helios protein in CD4^+^ T cells in a VDR-dependent manner. Finally, we identified conserved candidate VDRE sequences in the *Ikzf2* and *Bhmtl* gene promoters. Based on these observations, we suggest that proliferating, myelin peptide-specific CD4^+^ T cells have an intrinsic mechanism whereby 1,25(OH)_2_D_3_-VDR signaling increases Helios and BHMT1, prevents HCY accumulation, replenishes MET, maintains global DNA methylation, promotes CD4^+^Helios^+^FoxP3^+^ Treg cell dominance, and reverses established EAE (Figure 1B,C).

The demonstration that 1,25(OH)_2_D_3_ rapidly increases Helios and promotes CD4^+^Helios^+^FoxP3^+^ Treg cell dominance is significant in the context of emerging evidence that Treg cells, originally named for their immunoregulatory role, also have myelin regenerative functions [204206]. Oligodendrocyte differentiation and remyelination were impaired in Treg-deficient mice and these defects were rescued by adoptive Treg cell transfer. Moreover, in brain slice cultures, the Treg-derived mediator, CCN3, promoted oligodendrocyte progenitor cell differentiation and myelination.

The discovery that 1,25(OH)_2_D_3_ rapidly increased flux through the MET cycle is significant in the context of emerging evidence that the MET cycle is impaired in MS patients [207]. The *BHMT* gene sequence is conserved in sea urchins, amphibians, reptiles, birds and mammals, attesting to the evolutionary importance of HCY recycling [208]. In mammals, BHMT supports neurological health. Betaine supplementation prevented HHcy, neurological dysfunction, and high mortality in humans and mice with genetic methyltetrahydrofolate reductase deficiency blocking the MTR pathway [209,210]. In sharp contrast, folate supplementation did not prevent HHcy and neurological dysfunction in mice with a genetic *Bhmtl* deficiency blocking the BHMT pathway [211]. Given the neuro-protective effects of betaine, it is significant that betaine levels were decreased in MS gray matter, parietal, and motor cortex regions compared to samples from healthy controls [212]. These investigators observed BHMT protein in neurons, and reported that decreased betaine correlated with diminished histone H3 trimethylation and mitochondrial respiratory defects in these neurons. In summary, compelling and varied data from rodent and human studies is converging to emphasize the importance of vitamin D_3_, betaine and BHMT1 for neurological health.

HHcy has been causally linked to MS, particularly in men [213–216]. In MS patients, HHcy correlated with brain atrophy [217], cognitive impairment [218], depression [219], and surprisingly, with β-interferon use [220]. The HHcy observed in MS patients could not be explained by inadequate vitamin B_6_, vitamin B_12_ or folate to support the MTR pathway [213]. Mechanistically, HHcy promoted neuronal cell excitotoxicity, oxidative stress, mitochondrial damage, and cell death [221,222]. Others have suggested that HHcy and widespread methyltransferase impairment may be a key to MS susceptibility [223]. Collectively, our EAE modeling data, together with observations on the association between HCY and MS brain atrophy, suggest that diminished 1,25(OH)_2_D_3_-VDR signaling in CD4^+^ T cells, neurons, or glial cells might decrease BHMT1 activity, reduce SAM availability, increase HCY, and ultimately cause neuronal cell toxicity.

A vital question in MS research is why the CD4^+^FoxP3^+^ Treg cells from MS patients are functionally defective and unstable [20]. In answer to this question, we envisioned 1,25(OH)_2_D_3_ as a hormonal switch between two bi-stable T cell states, promoting anti-inflammatory gene expression and dampening pro-inflammatory gene expression to restore a dominant, self-tolerant CD4^+^ T cell state [24]. The human *FOXP3* gene and its murine ortholog harbor three conserved VDREs in a region that must undergo DNA demethylation for stable FOXP3 expression and Treg lineage commitment [180,224]. Liganded VDR binding to these VDREs enabled other nuclear factors to activate *FOXP3* transcription. Helios is one of the nuclear factors that activates *FOXP3* transcription [16,201]. We hypothesize that 1,25(OH)_2_D_3_-VDR signaling through VDRE sequences in the *IKZF2* and *FOXP3* promoter regions stabilizes FOXP3 expression and Treg lineage commitment, restoring expression of the Treg cell signature proteins [225]. We also hypothesize that 1,25(OH)_2_D_3_-VDR signaling promotes DNA methylation of CpG sites in the human and murine *112* and *Ifng* genes for epigenetic inhibition of IL-2 and IFN-γ production. Inhibition of IL-2 production is essential for CD4^+^FoxP3^+^ Treg cell stability [226,227] and inhibition of IFN-γ production is essential to downregulate Th1 cells. By upregulating Helios and FOXP3 and downregulating IL-2 and IFN-γ, 1,25(OH)_2_D_3_-VDR signaling could cause a switch from a dominant pro-inflammatory to a dominant anti-inflammatory CD4^+^ T cell state [24]. Beyond functioning as a hormonal switch, our data further suggest that increased 1,25(OH)_2_D_3_-VDR signaling in CD4^+^ T cells could be critical for the epigenetic marks that suppress inappropriate gene expression.

Returning to the observation that CD4^+^ T cells from MS patients showed DNA hypomethylation and inappropriate *HLA-DRB1* expression [29,46], it is of paramount importance that the promoter region of the European-derived DRB1*1501 allele reportedly harbors a VDRE [43]. We found no reports of how this putative VDRE may function in human CD4^+^ T cells. However, in the context of the vitamin D, Treg cell, and DRB1*1501 epigenetic hypothesis of MS risk (Figure 1D), it seems plausible that reduced 1,25(OH)_2_D_3_-VDR signaling and MET cycle flux could decrease DNA methylation within the MHC class II DRB1*1501 MS risk allele, causing inappropriate expression and dysregulation within the CD4^+^ T cell compartment. If 1,25(OH)_2_D_3_-VDR signaling through the putative VDRE in the European-derived DRB1 *1501 allele were to increase DNA methylation and reduce its expression in MS patient CD4^+^ T cells, then one could suggest that these epigenetic marks are dynamic in post-natal life rather than fixed in gestation, which is critically important information for MS prevention efforts.

It would also be profoundly interesting to learn whether the putative VDRE identified in the European DRB1*1501 allele exists in the African DRB1*1501 allele. The European DRB1*1501 alleles had a common amino acid sequence and conferred a 3-fold greater MS risk than the African DRB1*1501 alleles, which showed amino acid sequence diversity in regions related to peptide antigen presentation [49]. The sequencing data suggest the European DRB1*1501 allele arose after the human migration out of Africa and underwent positive selection for a European antigen, whereas the African DRB1*1501 alleles continued to undergo mutation and selection for African antigens. Considering the 3-fold difference in MS risk between the European and African alleles, these questions quite likely go to the heart of MS molecular etiology, probing the fabric of susceptibility and resistance genes, gender, and environmental interactions to understand this complex neurodegenerative disease.

Next, we consider vitamin D_3_ and EAE demyelinating disease prevention study results. No studies have yet evaluated vitamin D_3_ supplementation in the context of MS prevention, but considerable information is available from EAE prevention studies. Vitamin D_3_ supplementation given to intact weanling female mice one month prior to EAE induction and continuing to the end of the study reduced EAE incidence and severity [81]. However, this protocol provided no benefit to male mice. Others confirmed this observation [84]. A similar protocol inhibited EAE induction in weanling female rats, but vitamin D_3_ supplementation given to adult female rats age >12 weeks, or only during the pre- and early post-natal period, did not inhibit EAE induction [83]. These and other related data have been reviewed [2,24].

A recent pathway analysis of differentially-expressed genes revealed downregulation of pro-inflammatory genes in splenic T cells from vitamin D_3_ supplemented females but not males, consistent with the EAE inhibition data in C57BL/6 mice [84]. These patterns were not observed in the EAE-resistant PWD/PhJ strain. In B6. Chr^PWD^ consomic strains, vitamin D_3_ supplementation influenced EAE induction in a sex- and genotype-dependent manner, sometimes diverging from the effects observed in C57BL/6 females. These results suggest unknown factors influence the outcome of vitamin D_3_ supplementation on EAE in rodents.

Transcriptome, methylome, and pathway analyses have also been deployed to investigate the impact of supplementary vitamin D_3_ on EAE induction [194]. Compared to un-supplemented controls, the CD4^+^ T cells harvested from peripheral lymph nodes of vitamin D3-supplemented animals a week after myelin peptide immunization had reductions in the Jak/Stat, Erk/Mapk, and Pi3K/Akt/mTor signaling pathways that are critical for T-cell activation and differentiation into Th1 and Th17 cells. Notably, vitamin D_3_ supplementation induced gene expression changes in the orthologs of many candidate MS risk genes and myelin-reactive T cell signature genes, supporting the hypothesis that it could modulate MS risk.

Very recently, we reported that CD4^+^ T cells acquire 1,25-(OH)_2_D_3_ from cells in lymphoid tissues and inflamed sites by a paracrine mechanism (Figure 1A) [31]. These experiments are described below. In the course of these studies, we searched for novel impacts of vitamin D_3_ supplementation on CD4^+^ T cells in the EAE prevention model. Humans with CTLA4-inactivating mutations have an incompletely penetrant phenotype of impaired CD4^+^FoxP3^+^ Treg cell CTLA-4 expression and function, hyperactive effector CD4^+^ T cells, and a complex immune dysregulation syndrome [228,229]. In addition, myeloid cell 1,25-(OH)_2_D_3_ synthesis enhanced human CD4^+^ T cell CTLA-4 expression in vitro [160,175]. Based on these reports, we theorized that vitamin D_3_ supplementation might increase CD4^+^ T cell CTLA-4 expression in vivo. Vitamin D_3_ supplemented female mice were immunized to induce EAE and CNS-infiltrating CD4^+^ T cells were collected and analyzed at the peak of disease [31]. As expected, vitamin D_3_ supplementation inhibited EAE induction, and an increase in the proportion of CNS-infiltrating CD4^+^CD44^high^ Tconv cells and CD4^+^FoxP3+ Treg cells expressing CTLA-4 correlated with EAE inhibition. These changes were not present in vitamin D3-supplemented females with myeloid cell-specific *Cyp27b1* gene targeting. Inspection of a highly conserved 200 bp region near the mouse *Ctla4* gene transcription start site revealed a VDRE-type sequence that was 93% conserved in the human *CTLA4* promoter. These results are the first to suggest that vitamin D_3_ reduces the risk of CNS tissue injury partially through a mechanism involving microglial cell 1,25-(OH)_2_D_3_ production, paracrine signaling to CNS-infiltrating CD4^+^ Tconv and Treg cells, and CTLA-4 immune checkpoint activation.

### Vitamin D and Estrogen Synergy in CD4^+^ T Cell Regulation

MS incidence has tripled since 1950 [59]. The incidence trend is female-biased, specific for relapsing-remitting disease, and most evident at high latitudes, although recently it has also emerged in low latitude regions. The trend’s scope, magnitude, and rapidity implicate increasing exposure to a non-genetic risk factor is the driver. Noting that life style changes have fueled a global increase in the incidence of vitamin D deficiency correlating temporally with the global increase in the incidence of MS, we have proposed that there exists a cause-effect relationship between these two trends [192]. We previously reviewed the MS incidence data, the evidence for a global rise in vitamin D deficiency, sex-based differences in MS, and estrogen’s mechanisms in demyelinating disease [59]. The female to male MS incidence ratio rises dramatically from 1.6 before puberty to 3.6 after puberty, prompting the hypothesis that pubertal changes “must be related...to the acquisition of the disease” in females [230]. The authors noted this change at puberty “is so highly significant that it must be related … to the acquisition of the disease” in females. Further, they suggested an “endocrine explanation for the unbalanced sex ratio” and a mechanism, estrogen “acting on T lymphocyte subsets”. Of note, an article by Voskuhl et al. in this volume summarizes evidence for estrogen’s neuro-protective effects in MS patients. Here we summarize studies probing synergy between the vitamin D system and 17p-estradiol (E2) in CD4^+^ T cell regulation.

To study vitamin D system and E2 interactions, ovariectomized (OVX) adult females were compared to OVX females given estrus-or diestrus-level E2 implants before EAE disease induction (OVX/E2-replete). Unlike pregnancy-level E2, the diestrus-level E2 did not inhibit EAE independently of supplementary vitamin D_3_. The OVX surgery eliminated and diestrus-level E2 replacement restored the female-specific, vitamin D_3_-mediated protective effects [81,192]. Compared to OVX females, the OVX/E2-replete females had fewer *Ifng, T-bet, Il17, ROR-γt* transcripts indicative of CD4^+^ Th1 and Th17 cells in the CNS [192,231,232]. CD4^+^ T cell-specific *Esrl* gene ablation eliminated E2 protective actions indicating ERα regulation of CD4^+^ T cell function was a core protective mechanism [233].

Further research revealed a cooperative interaction between 1,25-(OH)_2_D_3_ and E2 in CD4^+^ T cells that drove Helios^+^FoxP3^+^ Treg cell differentiation [81,176,192]. Compared to OVX females, spinal cord tissue from the OVX/E2-replete and SHAM females had 7.5-fold more 1,25-(OH)_2_D_3_, ~32-fold more *Vdr* transcripts, and significantly fewer *Cyp24al* transcripts. However, the serum 1,25-(OH)_2_D_3_ levels did not differ between the OVX and OVX/E2-replete mice. Thus, de novo 1,25-(OH)_2_D_3_ synthesis occurred in the CNS of vitamin D_3_-supplemented mice. In the CNS of the OVX/E2-replete mice, E2 increased and prolonged 1,25-(OH)_2_D_3_-VDR signaling by promoting *Vdr* gene transcription and inhibiting *Cyp24al* gene transcription. Similarly, in myelin-reactive CD4^+^ T cells in vitro, E2 silenced *Cyp24a1* gene transcription and increased *Vdr* gene transcription >5-fold. These observations suggested that E2 might inhibit EAE through a CD4^+^ T cell-specific, VDR-dependent mechanism.

To test this hypothesis, we evaluated E2-mediated inhibition of EAE induction in female mice with T cell-specific *Vdr* gene targeting [176]. The E2 increased the number of CD4^+^Helios^+^FoxP3^+^ Treg cells in the CNS by 2-fold, and decreased the encephalitogenic CD4^+^ T cells in the CNS by 80% in a VDR-dependent manner. In summary, there appears to be a cooperative amplification loop within CD4^+^ T cells wherein 1,25-(OH)_2_D_3_ enhances E2 biosynthesis, and the E2 downregulates *Cyp24a1* gene transcription and increases *Vdr* gene transcription to promote CD4^+^ T cell responsiveness and CD4^+^Helios^+^FoxP3^+^ Treg cell development. If a similar cooperative interaction exists in women, a decline in vitamin D_3_ status could cause a breakdown in T cell self tolerance and a rise in MS incidence.

### Vitamin D Signaling and Glial Cell Regulation

Microglial cell activation is the earliest biomarker of MS lesion formation [234]. Microglia are specialized myeloid lineage cells seeded into the developing CNS during embryogenesis where they promote synapse formation and refinement, oligodendrogenesis, and myelin synthesis [235]. Quiescent microglia monitor the parenchymal environment for pathogens, cytokines, chemokines, neurotransmitters, and neuromodulators [236]. When microglia detect pathogens or pro-inflammatory signals, they become activated, proliferating cells expressing high levels of MHC class II molecules and co-stimulatory molecules, and producing chemokines and cytokines that enable them to recruit and activate CD4^+^ T cells efficiently [237].

We demonstrated highly localized 1,25-(OH)_2_D_3_ synthesis in the inflamed rodent CNS [81]. In vitro, the activated rodent microglial cells produced 1,25-(OH)_2_D_3_, expressed *Vdr* transcripts, and responded to 1,25-(OH)_2_D_3_ by decreasing *Ccl3* (MIPIα), *Il6,Il12, Tnf* (TNFα), and *Nos2* (iNOS) gene transcription in an IL-10-and SOCS3-dependent manner [238]. To test the hypothesis that activated microglia produce 1,25-(OH)_2_D_3_ in vivo for autocrine and paracrine signaling, we disrupted the *Cyp27b1^0^* gene in myeloid lineage cells (M-*Cyp27b1^0^* strain) without disrupting it in the kidney [31]. The M-*Cyp27b1^0^* mice grew and reproduced normally, unlike the global *Cyp27b1* gene-targeted mice which show growth retardation, hypocalcemia, and poor bone mineralization [239]. In the female WT mice but not the *M-Cyp27b1^0^* mice, supplementary vitamin D_3_ inhibited EAE induction. Thus, a functional *Cyp27bl* gene in the microglia was essential for vitamin D_3_-mediated inhibition of EAE disease induction (Figure 1A).

The oligodendrocyte’s myelin membrane undergoes continuous remodeling and repair [106]. In RRMS disease, the demyelinating lesions sometimes undergo myelin repair, enabling some recovery of neurological function. However, in progressive MS disease, the myelin repair mechanisms fail and neurological function is irreversibly lost. Accordingly, identifying mechanisms that promote myelin repair is a critical research goal.

Dissecting remyelination mechanisms in MS patients is difficult because myelin damage and repair occur concurrently, but animal modeling has greatly advanced our understanding of these mechanisms [240]. Three toxins have been used to induce demyelination in rodents, cuprizone, lysolecithin, and ethidium bromide. When the toxin is discontinued, a synchronous and predictable spatiotemporal process of remyelination follows. The EAE model has also been used to study remyelination. In these models, 1,25-(OH)_2_D_3_ and vitamin D_3_ are widely reported to promote remyelination within demyelinated lesions, in part by stimulating OPC differentiation into mature, myelinating oligodendrocytes.

Early imaging studies demonstrated that rat oligodendrocytes expressed the VDR and responded to 1,25-(OH)_2_D_3_ by increasing *Vdr, Ngf* (nerve growth factor), and *Trkb* (low affinity NGF receptor) transcripts [191] (Table 5). The VDR protein has been observed in the nuclei of rat oligodendrocytes in the demyelinated lesions undergoing remyelination [241]. Other imaging studies detected the VDR and CYP27B1 proteins in human glial cells [242]. These findings prompted the hypothesis that 1,25-(OH)_2_D_3_-VDR autocrine and paracrine signaling may support myelination in the brain (Figure 2E).

The effect of supplementary vitamin D_3_ on remyelination has been evaluated in rodent demyelinating disease models. We observed that vitamin D3-supplemented females were protected from demyelination and clinical EAE signs compared to un-supplemented females [81]. Similarly, in a toxin demyelinating disease model, vitamin D3-supplemented mice had less microglia activation, macrophage infiltration, and demyelination compared to un-supplemented mice [248]. Finally, a new rodent study demonstrated that vitamin D3-supplemented mice had less toxin-induced demyelination and improved motor function that correlated with increased neural stem cell proliferation, migration into the lesion site, and differentiation into mature, myelin-forming oligodendrocytes [249].

We administered 1,25-(OH)_2_D_3_ to mice with moderately severe EAE disease to initiate a synchronous process of recovery and observed remarkable remyelination of spinal cord demyelinated lesions three days later [250]. Similarly, in a toxin demyelinating disease model, administering 1,25(OH)_2_D_3_ increased the number of myelinating oligodendrocytes and the extent of remyelination compared to placebo controls [251]. Another research group demonstrated 1,25-(OH)_2_D_3_-mediated enhancement of oligodendrocyte differentiation in vitro and in the EAE treatment model [195,243]. Moreover, inhibiting VDR signaling blocked remyelination in toxin-induced demyelinated lesions [241]. These investigators also observed high VDR protein levels in the oligodendrocytes, astrocytes, and microglia within the demyelinated lesions of postmortem brain sections from MS patients. Collectively, the emerging evidence on 1,25-(OH)_2_D_3_-VDR paracrine signaling in the CNS suggests supplementary vitamin D_3_ would prevent or limit damage to the myelin sheath, while a short course of 1,25-(OH)_2_D_3_ treatment would promote remyelination of demyelinated lesions (Figure 2E).

## CONCLUSIONS AND PERSPECTIVES

We have summarized emerging knowledge on particular aspects of MS molecular etiology with the ultimate goal of reducing the impact of MS and perhaps other complex neurodegenerative diseases. We focused on mechanistic interactions between susceptibility and risk genes, lipid metabolism and obesity, and vitamin D and estrogen to better understand the possible causes of (i) myelin sheath destabilization and fragmentation, and (ii) distortion in the balance between pathogenic myelin-reactive CD4^+^ Th17 cell and protective CD4^+^ Treg cells. We hypothesize that myelin destabilization and pathogenic myelin-reactive CD4^+^ Th17 cell dominance develop simultaneously during the MS prodromal period, a quietly smoldering inflammation fueled by obesity and vitamin D insufficiency in the context of genetic susceptibility.

Genetic susceptibility to MS centers on the MHC class II DRB1*1501 allele, but epistatic interactions with resistance alleles and environmental factors modify its capacity to yield an MS phenotype. Among environmental factors, genetically-or environmentally-determined vitamin D_3_ deficiency is the major, non-transmissible component that acts at the population level in a female-biased manner to determine MS risk. Female adolescent obesity is also firmly linked to MS risk, particularly in DRB1*1501 carriers. Obesity and vitamin D_3_ deficiency interact, since obesity reduced circulating 25-(OH)D_3_ levels, low 25-(OH)D_3_ levels diminished 1,25-(OH)_2_D_3_-mediated suppression of leptin production, and leptin signaling blocked *Cyp27b1* gene expression and drove CD4^+^ Th17 cell development over CD4^+^ Treg cell development. The temporal correlation between the global rise in obesity, vitamin D deficiency and female MS incidence, and the mechanistic data summarized in this review suggest these trends are causally related.

At its core, MS is a demyelinating disease. The myelin sheath is crucial for rapid nerve signal transmission. Myelinating oligodendrocytes form spiral, multilamellar myelin sheaths that thicken at the innermost membrane and thin nondestructively at the outermost membrane to facilitate remodeling, plasticity, and repair throughout life. Myelinating oligodendrocytes continuously biosynthesize lipids for myelin assembly and repair. These cells express high levels of the SCD enzymes that catalyze the rate-limiting step in nervonic acid biosynthesis. Nervonic acid is the major FA component of brain sphingomyelin, sulphatides, and cerebrosides. It is essential for myelin membrane uniformity, integrity, and stability. Vitamin D deficiency depressed SCD activity in rat peripheral tissues. We hypothesize that vitamin D deficiency similarly depresses SCD activity in oligodendrocytes, contributing to nervonic acid depletion, myelin membrane instability, demyelination, and remyelination failure.

At its core, MS is also an inflammatory disease. The 1,25-(OH)_2_D_3_ and the VDR transduce UVB photon signals into biological mechanisms of gene regulation. By targeting the *Cyp27b1* gene in myeloid-lineage cells and the *Vdr* gene in CD4^+^ T cells, we demonstrated that CNS inflammation stimulated myeloid cells to produce 1,25-(OH)_2_D_3_ for autocrine signaling to other myeloid cells and paracrine signaling to CD4^+^ T cells. The microglia responded to 1,25-(OH)_2_D_3_-VDR signaling by increasing IL-10 production and decreasing chemokines, pro-inflammatory cytokines, and CD86 costimulatory molecule expression. These changes diminished their ability to recruit and activate CD4^+^ T cells. The CD4^+^ T cells responded to 1,25-(OH)_2_D_3_-VDR signaling by swiftly increasing BHMT1 activity and flux through the MET cycle. These changes removed HCY and provided SAM for epigenetic regulation of gene expression. Subsequently, global DNA methylation increased, transcription of CD4^+^ Treg cell signature genes under VDRE control increased *(Ikzf2, Foxp3, Ctla4, Il10)*, and transcription of CD4^+^ Th17 cell signature genes under epigenetic control decreased *(Il17, Il22, Ifng).* Collaboration between E2 and 1,25-(OH)_2_D_3_ in female CD4^+^ T cells heightened the effects of 1,25-(OH)_2_D_3_-VDR signaling when E2 was present and undermined them in its absence. Collectively, the gene expression changes prevented toxic HHcy, maintained global DNA methylation, and promoted CD4^+^Helios^+^FoxP3^+^ Treg cell dominance. In this way, autocrine and paracrine 1,25-(OH)_2_D_3_-VDR signaling diminished the impact of demyelinating disease.

Scientists aiming to reduce the impact of MS and perhaps Alzheimer’s disease and Parkinson’s disease must consider a daunting complexity of genetic, epigenetic, hormonal, nutritional, geographic, and cultural influences. The concept of tissue breakdown and inflammatory responses occurring simultaneously during a prodromal period which we hypothesize in MS pathogenesis can also be applied to Alzheimer’s and Parkinson’s disease pathogenesis. Probing deeply into molecular mechanisms, investigating the inter-relatedness of susceptibility and resistance genes, gender, and environmental exposures, and analyzing potential nutritional, behavioral, and hormonal interventions could yield the insights needed to quench these quietly smoldering neurodegenerative processes and spare individuals from neurodegenerative disease.

## Figures and Tables

**Table 1.** Fatty acids in brain sphingolipids: MS patients and healthy controls ^a^.

Fatty acid	Sphingomyelin		Cerebrosides		Sulfatides	
	Normal	MS	Normal	MS	Normal	MS
Saturated						
16:0, palmitic	7.4 ± 2.1	11.2 ± 4.4*	6.5 ± 2.5	11.8 ± 6.8*	13.1 ± 4.3	17.1 ± 2.8*
18:0, stearic	25.4 ± 2.4	34.3 ± 5.6***	9.2 ± 2.0	13.2 ± 1.5***	9.5 ± 2.8	12.6 ± 2.4*
24:0, lignoceric	6.6 ± 0.9	6.5 ± 1.9	10.7 ± 1.6	10.9 ± 2.9	9.8 ± 1.7	12.1 ± 1.7*
Total	48.2 ± 2.5	59.3 ± 7.5**	38.2 ± 4.9	47.6 ± 4.2***	41.9 ± 4.6	52.0 ± 6.1**
Monounsaturated						
18:1, oleic	3.5 ± 1.2	4.7 ± 3.4	3.3 ± 1.0	4.4 ± 4.3	3.7 ± 1.1	4.2 ± 1.7
24:1, nervonic	36.3 ± 2.5	25.7 ± 5.7***	40.3 ± 5.7	31.0 ± 5.3***	36.2 ± 3.7	28.1 ± 4.8***
Total	51.8 ± 2.5	40.7 ± 7.5**	61.8 ± 4.9	52.4 ± 4.2***	58.1 ± 4.6	48.0 ± 6.1**

a Brain white matter fatty acid analysis [133]. The data are percentages of total lipid weight. Values are mean ± S.D. for 9 individuals per group. The significance of differences between the MS and control samples is indicated;

* *P* < 0.05,

** *P* < 0.01,

*** *P* < 0.001.

**Table 2.** Myelin lipid composition: EAE rodents and healthy controls ^a^.

Fatty acid	Healthy control			EAE		
	External face (%)	Cytoplasmic face (%)	Bilayer total (%)	External face (%)	Cytoplasmic face (%)	Bilayer total (%)
Cholesterol	22.4	10.6	32.9	25.8	12.1	37.9
Phosphatidylcholine	12.1	8.7	20.8	8.9	6.5	15.4
Phosphatidylserine	0.7	2.4	3.1	4.7	2.4	7.1
Cerebroside sulfatide	6.4	0	6.4	3.8	0	3.8
Sphingomyelin	2.8	2.1	4.9	0.9	0.7	1.6

a Brain myelin lipid composition [141]. The data are percentages of total lipid weight.

**Table 3.** Fatty acids in brain sphingolipids: EAE rodents and healthy controls^a^

Fatty acid	Healthy control	EAE
Saturated		
16:0, palmitic	39.48	35.14*
18:0, stearic	18.85	18.06
Monounsaturated		
18:ln9c, oleic	18.19	20.30
18:ln9t, elaidic	5.92	8.16*
24:ln9, nervonic	1.91	not detected
Polvunsaturated		
C20:4n6, arachidonic	1.74	5.56*

a Brain white matter fatty acid analysis [137]. The data are percentages of total lipid weight. Values are mean for three experiments, one mouse per group. The significance of differences between the EAE and control samples is indicated;

* *P* < 0.05.

**Table 4.** l,25-(OH)_2_D_3_ regulation of CD4^+^ T cell gene expression ^a^.

GENE	SPECIES	REFERENCES
***Enhanced gene expression***		
Bhmtl *	mouse	[32]
*CCR2*	human	[170]
Ctla4*	mouse	[31,171]
CTLA4*	human	[170,172–175]
*CXCR3*	human	[170]
Cyp24al*	mouse	[176]
CYP24A1*	human	[177,178]
*CYP27B1*	human	[177]
Foxp3*	mouse	[82,171,176,179]
FOXP3*	human	[170,172–174,179–182]
Ikzf2*	mouse	[32,82,176]
*Il10*	mouse	[171,173,179,183,184]
*IL10*	human	[170,174,179,185–187]
*IL10RA*	human	[170]
IL2RA*	human	[174,178]
*ITGA2*	human	[170]
*LAG3*	human	[170]
*MGAT1*	human	[188]
*PLCG1*	human	[189]
*Tgfb*	mouse	[171,190]
Vdr*	mouse	[176,191–193]
VDR*	human	[173,177]
***Diminished gene expression***		
*CCR6*	human	[170]
*Hiflα*	mouse	[194]
*Ifng*	mouse	[82,195]
*IFNG*	human	[170,172,177,186]
*Il17*	mouse	[82,173,184,190,193,195–199]
*IL17F*	human	[170,172,173,186,187]
*IL21*	human	[172]
*1122*	mouse	[173]
*IL22*	human	[170,184,187]
*IL26*	human	[170]
*Il23r*	mouse	[184]
*Rorc*	mouse	[184,190]
TAGAP*	human	[178]

a This table includes only protein-coding genes.

* The indicates the gene control regions include an established or postulated VDRE. Regulation of CD4^+^ T cell gene expression has been investigated in vitro and in vivo using experimental approaches that measure direct transcriptional regulation or indirect influences on gene expression (changes in cell differentiation, epigenetic marks, and non-coding RNAs). We refer the interested reader to the original reports for the experimental details and findings.

**Table 5.** l,25-(OH)_2_D_3_ regulation of oligodendrocyte gene expression ^a^.

GENE	SPECIES	REFERENCES
***Enhanced gene expression***		
*Bdnf*	mouse	[243]
*Cntf*	mouse	[243]
*Gdnf*	mouse	[243,244]
*Ngf*	mouse	[191,245]
Ngfr*	mouse	[244]
M/3	mouse	[243,246]
*Trkb*	mouse	[191,247]
Vdr*	mouse, human	[191,242,243,246]

a This table includes only protein-coding genes.

* The indicates genes with an established or postulated VDRE. Regulation of oligodendrocyte gene expression has been investigated in vitro and in vivo using experimental approaches that measure direct transcriptional regulation or indirect influences on gene expression (changes in cell differentiation, epigenetic marks, and non-coding RNAs). We refer the interested reader to the original report for the experimental details and findings.

## ABBREVIATIONS

1,25-(OH)_2_D_3_: 1,25-dihydroxyvitamin D_3_
24-hydroxylase: 1,25-dihydroxyvitamin D_3_ 24-hydroxylase
25-(OH)D_3_: 25-hydroxyvitamin D_3_
APC: antigen-presenting cells
BBB: blood-brain barrier
BDNF: brain-derived neurotrophic factor
BHMT1: betaine-homocysteine methyltransferase-1
BMI: body-mass index
CNS: central nervous system
CSF: cerebrospinal fluid
E2: 17β-estradiol
EAE: experimental autoimmune encephalomyelitis
FA: fatty acid
HCY: homocysteine
HHcy: hyper-homocysteinemia
HMG-CoAR: 3-hydroxy-3-methylglutaryl-coenzyme A reductase
LepR: leptin receptor
MBP: myelin basic protein
MET: methionine
MHC: major histocompatibility complex
MS: multiple sclerosis
MTR: methyltetrahydrofolate:homocysteine methyltransferase
MUFA: mono-unsaturated fatty acid
OPC: oligodendrocyte precursor cell
OR: odds ratio
OVX: ovariectomized
PLP: proteolipid protein
RORγt: retinoid receptor-related orphan nuclear receptor gamma-t
RRMS: relapsing-remitting multiple sclerosis
SAH: *S*-adenosyl-homocysteine
SAM: *S*-adenosyl-methionine
SCD: stearoyl-CoA (Δ9) desaturase
TCR: T cell receptor
TLR: Toll-like receptor
Th: T helper
Treg: T regulatory
SNP: single nucleotide polymorphism
SREBP: sterol regulatory element binding protein
UVB: ultraviolet B
VDR: vitamin D receptor
VDRE: vitamin D response element
